# A Natural Small Molecule Mitigates Kidney Fibrosis by Targeting Cdc42‐mediated GSK‐3β/β‐catenin Signaling

**DOI:** 10.1002/advs.202307850

**Published:** 2024-01-19

**Authors:** Xinrong Hu, Lu Gan, Ziwen Tang, Ruoni Lin, Zhou Liang, Feng Li, Changjian Zhu, Xu Han, Ruilin Zheng, Jiani Shen, Jing Yu, Ning Luo, Wenxing Peng, Jiaqing Tan, Xiaoyan Li, Jinjin Fan, Qiong Wen, Xin Wang, Jianbo Li, Xunhua Zheng, Qinghua Liu, Jianping Guo, Guo‐Ping Shi, Haiping Mao, Wei Chen, Sheng Yin, Yi Zhou

**Affiliations:** ^1^ Department of Nephrology The First Affiliated Hospital Sun Yat‐sen University NHC Key Laboratory of Clinical Nephrology Guangdong Provincial Key Laboratory of Nephrology Sun Yat‐Sen University Guangzhou 510080 China; ^2^ School of Pharmaceutical Sciences Sun Yat‐sen University Guangzhou 510006 China; ^3^ Institute of Precision Medicine The First Affiliated Hospital Sun Yat‐sen University Guangzhou 510080 China; ^4^ Department of Medicine Brigham and Women's Hospital and Harvard Medical School Boston MA 02115 USA

**Keywords:** Cdc42, GSK‐3β/β‐catenin, kidney fibrosis, natural small molecules

## Abstract

Kidney fibrosis is a common fate of chronic kidney diseases (CKDs), eventually leading to renal dysfunction. Yet, no effective treatment for this pathological process has been achieved. During the bioassay‐guided chemical investigation of the medicinal plant *Wikstroemia chamaedaphne*, a daphne diterpenoid, daphnepedunin A (**DA**), is characterized as a promising anti‐renal fibrotic lead. **DA** shows significant anti‐kidney fibrosis effects in cultured renal fibroblasts and unilateral ureteral obstructed mice, being more potent than the clinical trial drug pirfenidone. Leveraging the thermal proteome profiling strategy, cell division cycle 42 (Cdc42) is identified as the direct target of **DA**. Mechanistically, **DA** targets to reduce Cdc42 activity and down‐regulates its downstream phospho‐protein kinase Cζ(p‐PKCζ)/phospho‐glycogen synthase kinase‐3β (p‐GSK‐3β), thereby promoting β‐catenin Ser33/37/Thr41 phosphorylation and ubiquitin‐dependent proteolysis to block classical pro‐fibrotic β‐catenin signaling. These findings suggest that Cdc42 is a promising therapeutic target for kidney fibrosis, and highlight **DA** as a potent Cdc42 inhibitor for combating CKDs.

## Graphical abstract

The natural compound **DA**, screened out from 16 diterpenoids extracted from *Wikstroemia chamaedaphne*, alleviates kidney fibrosis by blocking fibroblast activation in the pro‐fibrotic environment. **DA** binds to Cdc42 and inhibits p‐PKCζ/p‐GSK‐3β axis, thereby promoting the ubiquitinated proteolysis of β‐catenin and suppressing its downstream pro‐fibrotic signals.

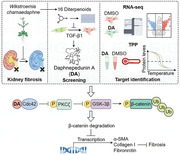

## 1 Introduction

Chronic kidney disease (CKD) is a major global public health concern affecting 10% of the general population with 1.2 million deaths annually.^[^ ^1^ ^]^ The progression of CKD to end‐stage kidney disease (ESKD) is driven by kidney fibrosis, which occurs in CKD of almost all etiologies and serves as the final common pathological pathway, leading to renal failure and death.^[^ ^2^ ^]^ The treatment of CKD has not gained substantial progress over the past decades.^[^ ^3^ ^]^ Clinically, dialysis and off‐label use of nonspecific medications are the only intervening choices, yet none of which alters kidney fibrosis or prevents the continued growth of mortality.^[^ ^4^ ^]^ Meanwhile, drugs undergoing trials against kidney fibrosis have limited efficacy in slowing disease progression and bring considerable side effects.^[^ ^5^ ^]^ For example, the anti‐fibrotic “star drug” pirfenidone (PFD) showed a transient benefit on renal function in phase II clinical trials but was discontinued among 14% of participants within 4 months by frequent adverse events including rash (10%) and gastrointestinal disorders (47%).^[^ ^6^ ^]^ In addition, given that the pharmacokinetic profile of PFD is dependent on renal function, the Food and Drug Administration (FDA) and the European Medicines Agency advise caution in using PFD in patients with reduced creatine clearance rate, a common manifestation of CKD, further limiting the clinical application of PFD in this regard.^[^ ^7^ ^]^ Thus, the development of anti‐kidney fibrosis agents with high efficacy and favorable safety profiles is urgently needed in CKD treatment.

Kidney fibrosis is a wound‐healing response to organ prolonged injuries, manifesting as the buildup of scar within the renal interstitium.^[^ ^8^ ^]^ The unremitting fibroblast activation including fibroblast‐to‐myofibroblast transformation (FMT), migration, and excessive deposition of extracellular matrix (ECM) proteins, has been recognized as a major event in the occurrence and progression of fibrosis.^[^ ^9^ ^]^ Among the many pro‐fibrotic factors, transforming growth factor‐β1 (TGF‐β1) is a well‐established master regulator of kidney fibrosis.^[^ ^10^ ^]^ In addition to the classic TGF‐β1/Smads signaling, TGF‐β1 can also activate other signaling cascades, including Wnt/β‐catenin, Notch, Hedgehog pathway, etc.^[^ ^11^ ^]^ As a result, these pathways integrate into a complex network downstream of TGF‐β1 to trigger fibroblast activation.^[^ ^12^ ^]^ Thus, targeting TGF‐β1 and its downstream molecules has always been considered an important direction and strategy for the development of anti‐CKD drugs.

Natural products (NPs) are evolutionarily selected for binding to specific biological macromolecules and thus represent a valuable source of “privileged structures” in drug discovery.^[^ ^13^ ^]^ So far, >50% of the FDA‐approved small‐molecule drugs were originated or inspired by NPs.^[^ ^14^ ^]^ In long‐term life practice, plenty of traditional herbal medicines have been used in the treatment of fibrosis‐related diseases, providing insights regarding efficacy and safety.^[^ ^15^ ^]^ However, their active constituents and exact action mechanisms are not determined. With the development of proteomic and chemobiological technologies, phenotype screening coupled with target fishing offers an efficient way to dissect these “mysterious” NPs. *Wikstroemia chamaedaphne* (*W. chamaedaphne*) is a shrub endemic in China. It has been widely used in folk medicines to treat edema, one of the most prominent manifestations of kidney‐related diseases, implying its enrichment of anti‐fibrotic NPs.^[^ ^16^ ^]^ As diterpenoids represent one of the most druggable NP classes and are enriched in *W. chamaedaphne*,^[^ ^17^ ^]^ in the present study, we launched a phenotypic screen based on the TGF‐β1‐activated renal fibroblast model to evaluate the main diterpenoids isolated from this plant. Among them, daphnepedunin A (**DA**) was screened out as the most potent inhibitor of kidney fibrosis both in vitro and in vivo. By integrating transcriptomics technology and thermal proteome profiling, we identified cell division cycle 42 (Cdc42) as the target protein of **DA**. Mechanistically, **DA** deactivates Cdc42 and then promotes β‐catenin proteolysis via phospho‐protein kinase Cζ (p‐PKCζ)/phospho‐glycogen synthase kinase‐3β (p‐GSK‐3β) signaling axis. Collectively, our study demonstrates that **DA** is a promising lead for the development of anti‐renal fibrotic agents and that Cdc42 is a hitherto unrecognized potential therapeutic new target for kidney fibrosis.

## 2 Results

### 2.1 Discovery of **DA** as a Potent Inhibitor of Renal Fibroblast Activation

**Figure 1:**
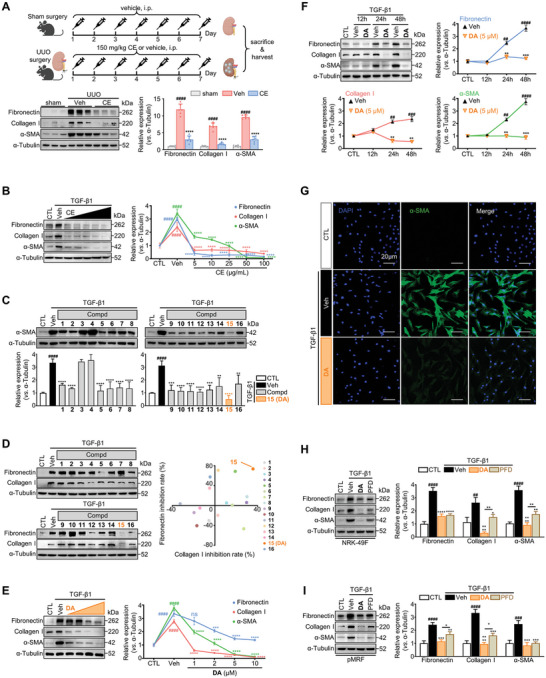
Identification of **DA** as a potent antifibrotic compound in TGF‐β1‐activated renal fibroblasts. A) Sham or UUO surgery was performed on C57BL/6J mice. Vehicle (Veh) or crude extract of *W. chamaedaphne* (CE, 150 mg kg^−1^) was intraperitoneally administered to mice daily for the consecutive 7 days after the surgery. Kidney homogenate samples were analyzed by western blotting (*n =* 6 per group) to quantify the protein levels of fibronectin, collagen I, and α‐SMA. B) Influence of CE on expression levels of fibrotic markers (fibronectin, collagen I, and α‐SMA). Immunoblotting analysis was carried out in TGF‐β1‐activated NRK‐49F cells treated with vehicle or indicated concentrations of CE for 48 h (*n =* 3 per group). C) The effects of diterpenoid 1–16 (**1**–**16**) on α‐SMA. Immunoblotting analysis was carried out in TGF‐β1‐activated NRK‐49F cells treated with vehicle or 10 µM compounds for 48 h (*n =* 3 per group). D) The inhibition of **1**–**16** on ECM (fibronectin and collagen I). The inhibition rate of each compound was calculated by the ratio of ECM levels in each group to Veh group [Compd/Veh (%)] (*n =* 3). E) Western blot analysis performed in TGF‐β1‐activated NRK‐49F cells treated with vehicle or indicated concentrations of **DA** for 48 h (*n =* 3 per group). F) The fibrotic protein levels were assessed in TGF‐β1‐activated NRK‐49F cells treated with vehicle or 5 µM **DA** at 12, 24, and 48 h (*n =* 3 per group). G) Immunostaining of α‐SMA (green) in NRK‐49F cells following 48 h of TGF‐β1 treatment. Scale bar = 20 µm. H, I) Immunoblot analysis of TGF‐β1‐stimulated NRK‐49F (H) and primary murine renal fibroblasts (pMRF) (I). Level of fibrotic proteins in **DA** or pirfenidone (PFD, 5 µM) treated cells (*n =* 3 per group). Control, CTL; vehicle, Veh. Data are presented as means ± SEM (A, B, C, D, E, F, H, and I). Data were analyzed using one‐way ANOVA followed by Bonferroni's multiple comparisons test. ^##^ *p <* 0.01, ^###^ *p <* 0.001, ^####^ *p <* 0.0001 compared with CTL group; * *p <* 0.05, ** *p <* 0.01, *** *p <* 0.001, **** *p <* 0.0001 compared with Veh group or between groups under the line.

In our ongoing efforts toward discovering biologically active anti‐fibrotic natural products,^[^ ^18^ ^]^ we observed that the crude extract (CE) of *W. chamaedaphne* significantly attenuated kidney fibrosis in mice. As shown in **Figure** **1**A, administration of 150 mg kg^−1^ of CE dramatically reduced α‐SMA and ECM (fibronectin and Collagen I) levels in mice received unilateral ureteral obstruction (UUO) surgery, a widely used animal model of kidney fibrosis.^[^ ^19^ ^]^ Since activated renal fibroblasts are well‐recognized culprits in kidney fibrosis, we then moved on to determine the effects of *W. chamaedaphne* on these cells by utilizing TGF‐β1‐stimulated rat kidney fibroblast normal rat kidney‐49F (NRK‐49F) as an in vitro model. In Figure 1B, TGF‐β1 (10 ng mL^−1^ for 48 h) stably induced the expression of α‐SMA and production of ECM in renal fibroblasts, while CE of *W. chamaedaphne* resulted in a dose‐dependent decrease in the expression of those fibrotic indicators starting at 5 µg mL^−1^, suggesting its potent activity to repress the activation of renal fibroblasts. To further trace the active constituents in CE, we performed a bioassay‐guided fractionation. CE was suspended in water and then successively partitioned with petroleum ether, ethyl acetate (EtOAc), and n‐butanol. Each fraction was tested for anti‐renal fibrosis activity, and the EtOAc fraction that displayed a promising activity was selected for further chemical investigation. Subsequent systematic purification of this fraction using various chromatographic methods led to the isolation of 16 diterpenoids (**1**–**16**) (Table S1, Supporting Information). These diterpenoids were then tested on activated fibroblasts to clarify their anti‐fibrotic potential. Interestingly, many of these compounds displayed similar but not identical capability to attenuate TGF‐β1‐induced α‐SMA expression (Figure 1C). Among them, compound **15**, referred to as daphnepedunin A (**DA**), showed the strongest activity, sharply down‐regulating the α‐SMA level by 3.5‐fold relative to the vehicle (veh) group. In terms of ECM, **DA** also exhibited the most outstanding inhibitory efficiency against fibronectin and collagen I among the candidate compounds (Figure 1D). Meanwhile, **DA** presented low toxicity to fibroblasts in MTS assay, with cell viability >90% at 10 µM (Figure S1A, Supporting Information). Therefore, considering both efficacy and safety, **DA** was selected as a promising lead for further study. This compound reduced the expression of fibrotic markers in a dose‐ and time‐dependent manner (Figure 1E,F), with striking activity at 5 µM for 48 h. Under this condition, the marked inhibition of myofibroblast activation was also confirmed by α‐SMA immunofluorescence staining (Figure 1G).

To evaluate the translational potential of **DA** in kidney fibrosis, we employed two established in vitro models to compare its efficacy with that of pirfenidone (PFD), an anti‐renal fibrotic agent currently undergoing phase II clinical trial.^[^ ^5^ ^]^ As **DA** at a concentration of 5 µM did not show detectable growth inhibition to NRK‐49F or primary murine renal fibroblasts (pMRF), thus, this concentration was applied for further studies (Figure S1B, Supporting Information). In comparison to PFD, **DA** displayed a much stronger inhibitory effect on TGF‐β1‐induced activation of NRK‐49F and pMRF, especially on the expression of collagen I and α‐SMA, under the same treatment conditions (Figure 1H,I). Similar results were also observed in comparison with other commonly used CKD medications, such as RAAS blockers valsartan (Val) or enalapril (Ena),^[^ ^20^ ^]^ in particular in reducing α‐SMA and ECM expression (Figure S2A, Supporting Information). Taken together, we demonstrate that **DA** is a predominant component of *W. chamaedaphne* to significantly inhibit renal fibroblast activation and ECM production.

### 2.2 **DA** Suppresses the Activation of Fibroblasts Derived from Epithelial‐Mesenchymal Transition of Renal Tubular Cells

**Figure 2:**
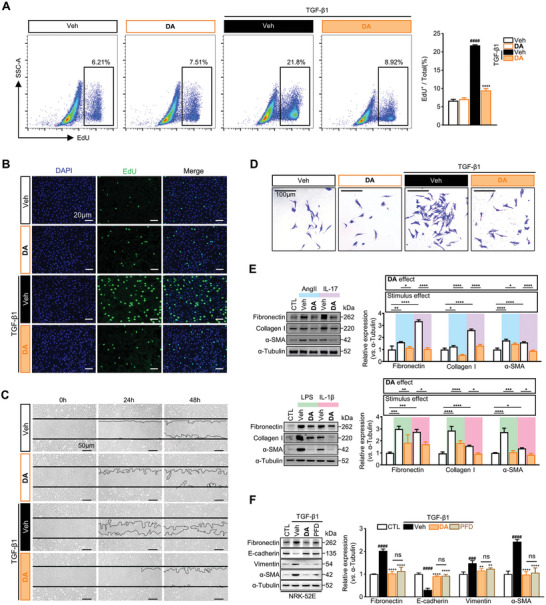
**DA** blocked the activation of renal fibroblasts and EMT of renal tubular cells. A, B) EdU assay was performed on TGF‐β1‐stimulated NRK‐49F cells. Representative flow cytometry (*n =* 3 per group) (A) and confocal images (B) showed that EdU positive ratio cells, scale bar, 20 µm. C) Microscopy images of the wound‐healing assay at different times. The solid lines showed the initial area without cells and the dashed lines indicated the margin of migrated cells. The scale bar represents 50 µm. D) Presentative microscopic images of pMRF cells migrating from the upper chamber to the lower chamber. Scale bar, 100 µm. E) Immunoblots showing the effects of **DA** on the fibrotic proteins in NRK‐49F cells incubated with AngII (10 µM), IL‐17 (50 ng mL^−1^), LPS (25 ng mL^−1^) or IL‐1β (10 ng mL^−1^) (*n =* 3 per group). F) Immunoblot analysis of TGF‐β1‐stimulated NRK‐52E cells (fibronectin, E‐cadherin, vimentin, and α‐SMA) in **DA** or pirfenidone (PFD, 5 µM) treated group (*n =* 3 per group). Control, CTL; vehicle, Veh. Data are presented as means ± SEM (A, E, and F) and were analyzed using one‐way ANOVA followed by Bonferroni's multiple comparisons test. ^###^ *p <* 0.001, ^####^ *p <* 0.0001 compared with CTL group; * *p <* 0.05, ** *p <* 0.01, *** *p <* 0.001, **** *p <* 0.0001 compared with Veh group or between groups under the line; ns, not significant.

Apart from differentiating into myofibroblasts and substantially producing ECM, activated renal fibroblasts exhibit enhanced proliferative and migratory properties, leading to renal fibrotic lesions.^[^ ^21^ ^]^ Hence, we further explored the effects of **DA** on these properties of activated fibroblasts. To this end, first, in the absence of TGF‐β1, we did not observe an obvious difference in fibroblast proliferation (EdU^+^) between **DA**‐ and vehicle‐treated groups, suggesting that **DA** had no effects on the basal proliferative ability of renal fibroblasts. TGF‐β1 stimulation promoted the expansion of proliferative cells (EdU^+^), whereas DA blocked such expansion (**Figure** **2**A,B). Wound healing assays yielded a similar conclusion. While **DA** did not affect the migratory capacity of quiescent fibroblasts, it blocked TGF‐β1‐induced fibroblasts migrating, as measured by wound closure rate (Figure 2C; Figure S2B, Supporting Information). In echoing these findings, transwell migration assays were performed with pMRF and showed that the migration of pMRF was readily alleviated by **DA** under the condition of TGF‐β1‐stimulation, but not in intact pMRF cells (Figure 2D; Figure S2C, Supporting Information). Collectively, these data reveal that **DA** robustly eliminates the proliferative and migratory properties of activated fibroblasts.

Given that various cytokines in the renal fibrotic microenvironment contribute to the activation of fibroblasts, we examined whether **DA** could block the pro‐fibrotic effects of these cytokines, such as angiotensin II (Ang II), interlukin‐17 (IL‐17), lipopolysaccharide (LPS), IL‐1β, etc.^[^ ^22^ ^]^ As illustrated in Figure 2E, **DA** predominantly attenuated the expression of α‐SMA and the ECM induced by these cytokines. Although myofibroblasts in the fibrotic kidney are mostly derived from fibroblasts, the hypothesis remains that they arise in part from renal tubular epithelial cells through epithelial‐mesenchymal transition (EMT).^[^ ^23^ ^]^ Thus, we used TGF‐β1‐stimulated renal tubular epithelial cells to investigate whether **DA** was able to prevent EMT. As expected, **DA** exhibited a similar inhibitory effect on EMT as PFD, as shown by the restoration of epithelial marker E‐cadherin and the decreases of mesenchymal markers (fibronectin, vimentin, and α‐SMA) (Figure 2F). Surprisingly, the drug effects of **DA** were even validated in TGF‐β1‐activated LX2 (human hepatic stellate cells, a key player in liver fibrosis^[^ ^24^ ^]^), which seems to provide hints that the anti‐fibrotic activity of **DA** may be universal across multiple fibrotic diseases (Figure S2D, Supporting Information).

### 2.3 **DA** Mitigates Kidney Fibrosis in UUO Mouse Model

**Figure 3:**
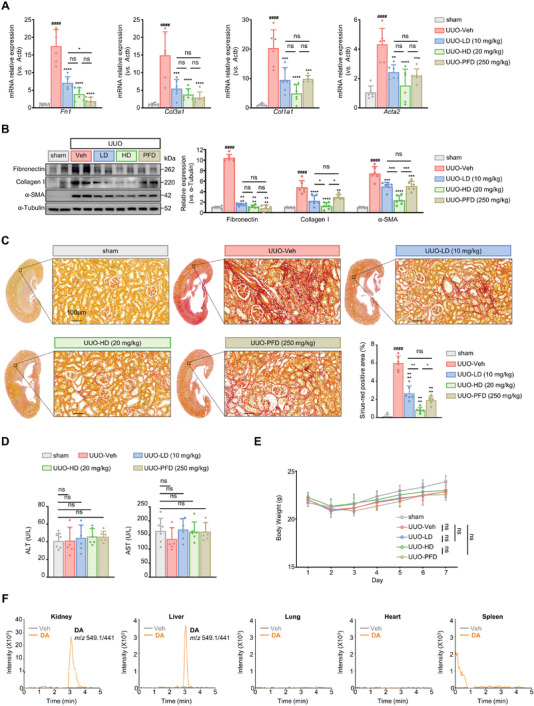
**DA** protected against kidney fibrosis in UUO mice. A) The transcript levels of fibronectin (*Fn1*), collagen III (*Col3a1*), collagen I (*Col1a1*), and α‐SMA (*Acta2*) in **DA** or PFD‐treated mouse kidneys were examined by q‐PCR 7 days after sham or UUO surgery. β‐actin (*Actb)* was used as the internal reference (*n* = 6 per group). B) The protein levels of fibronectin, collagen I, and α‐SMA in **DA** or PFD‐treated mouse kidney homogenate samples analyzed by SDS‐PAGE 7 days after sham or UUO surgery (*n* = 6 per group). C) Representative images of picrosirius red staining and quantitative analyses of **DA** or PFD‐treated mouse kidneys 7 days after sham or UUO procedures, scale bar, 100 µm (*n* = 6 per group). D) The levels of serum AST and ALT of **DA** or PFD‐treated mice measured at day 7 after the surgery. E) Mice body weights in the 7 consecutive days after sham or UUO surgery (*n* = 6 per group). F) Kidney, liver, lung, heart, and spleen from mice treated with vehicle or 20 mg kg^−1^ **DA** were collected. Tissue supernatants from their homogenates were analyzed by HPLC‐MS/MS. Vehicle, Veh; low dose, LD; high dose, HD; pirfenidone, PFD. Data are presented as means ± SEM (A, B, C, D, and E) and were analyzed using one‐way ANOVA (A, B, C, D) or two‐way ANOVA (E) followed by a Bonferroni's multiple comparisons test unless otherwise stated. ^####^ *p <* 0.0001 compared with sham group; * *p <* 0.05, ** *p <* 0.01, *** *p <* 0.001, **** *p <* 0.0001 compared with UUO‐Veh group or between groups under the line; ns, not significant.

To assess the anti‐fibrosis effect of **DA** in vivo, we employed the UUO mouse model, a well‐established model to study tubulointerstitial fibrosis. For that, both low‐dose (LD, 10 mg kg^−1^) and high‐dose (HD, 20 mg kg^−1^) of **DA** were administrated (daily, intraperitoneal (i.p.) injection) for 7 days after the surgery. Meanwhile, PFD (250 mg kg^−1^ daily i.p.) was utilized as the positive control.^[^ ^25^ ^]^ As expected, **DA** significantly down‐regulated the elevated mRNA levels of *Acta2* (encoding α‐SMA), *Col1a1*, and *Col3a1* (encoding collagens) and *Fn1* (encoding fibronectin), as well as the corresponding protein levels in kidneys from UUO mice, as compared to vehicle (**Figure** **3**A,B). Of note, the low‐dose **DA** group achieved a similar potency to PFD group (250 mg kg^−1^) in reducing these fibrotic regulators. Strikingly, high‐dose **DA** displayed much better efficiency than PFD group, supporting the strong anti‐kidney fibrosis activity of **DA**. Similar results were also observed in kidney collagen deposition detected with picrosirius red staining (Figure 3C). Importantly, **DA** administration did not induce overt systemic toxicity in mice, as indicated by the levels of serum liver enzyme (aspartate aminotransferase (AST) and alanine aminotransferase (ALT)) and the loss of body weight (Figure 3D,E).

The distribution of drugs into intended sites is vital for both increasing efficacy and reducing side effects.^[^ ^26^ ^]^ Thus, we adopted the widely used high performance liquid chromotagraphy‐tandem mass spectrometry (HPLC‐MS/MS) method to detect the amount in different organs.^[^ ^27^ ^]^ As shown in Figure 3F, in the kidney and liver, peaks of **DA** were found in the chromatograms, and **DA** appeared to be more enriched in the kidney. However, in lung, heart, and spleen, the **DA** peak was absent. Overall, these findings suggest that **DA** is a potent renoprotective agent that can alleviate tubulointerstitial fibrosis in vivo, with high efficacy and safety.

### 2.4 **DA** Reduces β‐Catenin Accumulation in Activated Fibroblasts

**Figure 4:**
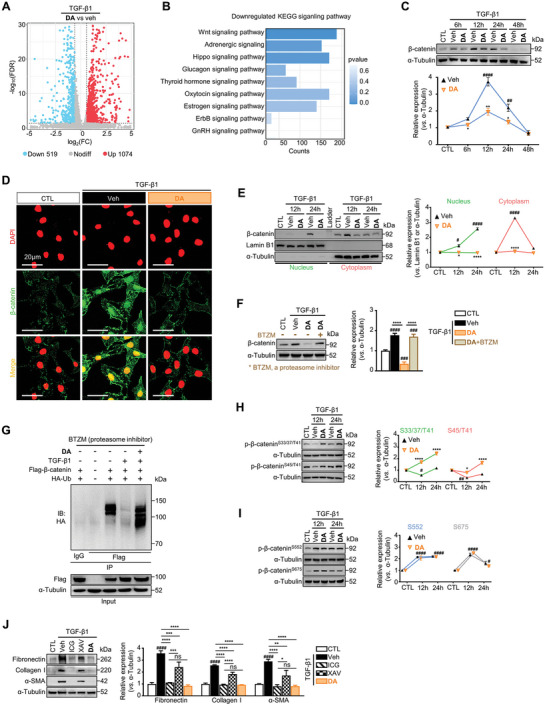
**DA** promoted the phosphorylation of β‐catenin at S33/37/45/T41 and targeted β‐catenin for ubiquitin‐proteasome degradation. A) Volcano plots depicting the differentially expressed genes between vehicle‐treated (veh) and **DA**‐treated groups. B) Barplot showing all the down‐regulated KEGG pathways termed signaling pathway. C) Immunoblot analysis of β‐catenin in TGF‐β1‐activated NRK‐49F treated with vehicle or **DA** at instructed times (*n =* 3 per group). D, E) The protein levels of β‐catenin in the nucleus and cytoplasm of NRK‐49F treated with vehicle or **DA**. Subcellular cytosolic and nuclear fractions of β‐catenin were analyzed by immunofluorescence (scale bar, 20 µm) (D) and western blot (*n =* 3 per group) (E). Green fluorescence signals represent β‐catenin proteins. Red signals represent the nucleus, scale bar: 20 µm. F) Immunoblot showing the protein levels of β‐catenin in cells treated with vehicle, **DA**, or **DA**+bortezomib (BTZM, 1 nM), a proteasome inhibitor (*n =* 3 per group). G) Effects of **DA** on β‐catenin ubiquitination. HA‐Ub was co‐transfected into NRK‐49F cells with Flag‐β‐catenin constructs in the presence of BTZM. Immunoprecipitation (IP) was performed by anti‐Flag beads or IgG beads and immunoblotting (IB) with anti‐HA antibodies. H, I) Protein levels of phosphorylated β‐catenin (p‐β‐catenin) at different amino acid residues including serine (S) 33, 37, 45, 552, 675, and threonine (T) 41 were assessed at 12 and 24 h in NRK‐49F cells. NRK‐49F cells were activated by TGF‐β1 and treated with vehicle or **DA** (*n =* 3 per group). J) Western blots performed on TGF‐β1‐activated NRK‐49F following the treatment of **DA** (5 µM), ICG001 (ICG, 5 µM), XAV939 (XAV, 5 µM) respectively (*n =* 3 per group). Control, CTL; vehicle, Veh; Ser, S; Thr, T. Data are presented as means ± SEM (C, E, F, H, I, and J) and were analyzed using one‐way ANOVA followed by a Bonferroni's multiple comparisons test. ^#^ *p <* 0.05, ^##^ *p <* 0.01, ^###^ *p <* 0.001, ^####^ *p <* 0.0001 compared with CTL group; * *p <* 0.05, ** *p <* 0.01, *** *p <* 0.001, **** *p <* 0.0001 compared with Veh group or between groups under the line; ns, not significant.

To elucidate the molecular mechanism underlying the anti‐fibrotic effect of **DA**, we performed RNA‐sequencing on TGF‐β1‐activated NRK‐49F cells treated with/without **DA**. The results showed that 1593 genes were differentially expressed between the two groups, with 1074 genes up‐regulated and 519 genes down‐regulated upon DA treatment (**Figure** **4**A). Unexpectedly, the Kyoto Encyclopedia of Genes and Genomes (KEGG) analysis of these genes revealed that **DA** exerted a significant effect on the Wnt signaling pathway, rather than the putative pro‐fibrotic TGF‐β/Smads signaling pathway (Figure 4B). Consistent with that, neither the expression of Smad2/3 nor their phosphorylation (p‐Smad2/3), key transcriptional effectors in TGF‐β/Smads pathway,^[^ ^28^ ^]^ was affected by **DA** (Figure S3A, Supporting Information). Therefore, β‐catenin, the central hub of the Wnt signaling cascade,^[^ ^29^ ^]^ was further focused. We observed that the expression of β‐catenin was subtle in quiescent fibroblasts, which could be up‐regulated by TGF‐β1 in 6 h and peaked around 12 h, followed by a gradual decrease. At each time point, **DA** blocked TGF‐β1‐induced upregulation of β‐catenin (Figure 4C). Next, we found that **DA** remarkably suppressed TGF‐β1‐stimulated β‐catenin cytoplasmic and nuclear accumulation, main features of Wnt/β‐catenin signaling activation as well as fibrotic gene transcription^[^ ^29^, ^30^ ^]^ (Figure 4D,E). Coherently, we detected an inhibitory effect of **DA** on β‐catenin levels induced by various stimuli in multiple renal fibroblasts and tubule cell models tested (Figure S3B–E, Supporting Information). These findings further supported that **DA** inhibited β‐catenin to combat kidney fibrosis.

Surprisingly, in contrast to its effect on suppressing TGF‐β1‐upregulated β‐catenin protein levels, **DA** increased the mRNA levels of β‐catenin (*Ctnnb1)* in renal fibroblasts (Figure S3F, Supporting Information). We thus hypothesized that **DA**‐mediated down‐regulation of β‐catenin might be due to protein degradation. To verify this, we employed a proteasome inhibitor bortezomib (BTZM)^[^ ^31^ ^]^ to block the ubiquitin‐proteasome system and observed that **DA** failed to block TGF‐β1‐induced β‐catenin upregulation upon BTZM (1 nM) administration, suggesting that **DA** reduces β‐catenin by enhancing its proteolysis (Figure 4F). Furthermore, we tended to examine the effects of **DA** on the ubiquitination modification of β‐catenin, utilizing renal fibroblasts co‐transfected with HA‐tagged ubiquitin (HA‐Ub) and Flag‐tagged β‐catenin. The results showed that TGF‐β1‐reduced β‐catenin ubiquitination could be rescued by **DA** treatment (Figure 4G). It has been well‐recognized that the ubiquitinated proteolysis of β‐catenin is tightly controlled by the phosphorylation status at specific sites, for instance, phosphorylation at Ser33/37/45 and Thr41 enhances β‐catenin degradation,^[^ ^32^ ^]^ while phosphorylation at Ser552/675 inhibits the proteolysis.^[^ ^33^ ^]^ Consistent with these reports, we found that TGF‐β1 significantly decreased the phosphorylation of β‐catenin at the Ser33/37/45/Thr41 sites, which could be antagonized by **DA** administration, thereby promoting β‐catenin for degradation (Figure 4H). By contrast, the phosphorylation of β‐catenin at Ser552/675 was not influenced by **DA** (Figure 4I). Therefore, these findings suggest that **DA** fosters the ubiquitinated proteolysis of β‐catenin via up‐regulating the phosphorylation of β‐catenin at Ser33/37/45/Thr41 residues.

Next, we also compared the anti‐fibrotic efficacy of **DA** with other known β‐catenin inhibitors ICG001 and XAV939.^[^ ^34^ ^]^ When these compounds were applied to renal fibroblasts at the same concentration, **DA** exhibited comparable activity to ICG001 in inhibiting fibrotic protein expression, but much more potent than XAV939 did (Figure 4J). Together, these observations suggest that the anti‐fibrotic activity of **DA** is mainly dependent on the blockade of the pro‐fibrotic β‐catenin signaling pathway.

### 2.5 **DA** Reduces β‐catenin Abundance in Fibrotic Kidney

**Figure 5:**
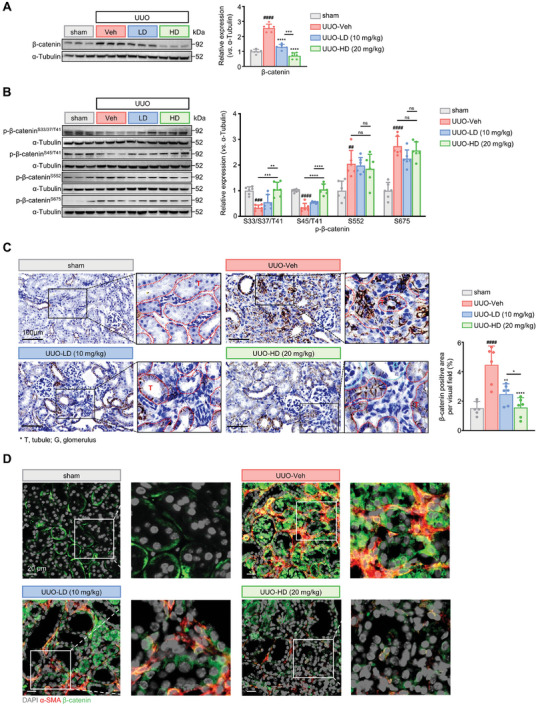
**DA** increased p‐β‐catenin (S33/37/45/T41) and reduced total β‐catenin in UUO kidneys. A, B) Kidney homogenate samples were analyzed by SDS‐PAGE (*n =* 6 per group). Protein levels of total β‐catenin (A), p‐β‐catenin (S33/37/45/552/675/T41) (B) were detected in each group 7 days after sham or UUO surgery (*n =* 6 per group). C) Representative images and quantitative analyses of the immunohistology results of β‐catenin in kidney tissue (*n =* 6 per group). Blue dashed lines outline the glomerulus and red dashed lines for the tubule. Scale bar, 100 µm. D) Representative images of immunofluorescent staining of UUO mice kidney samples. Expression and colocalization of α‐SMA (red) with β‐catenin (green) are shown. Grey signals represent the nucleus. Scale bar, 20 µm. Vehicle, Veh; low dose, LD; high dose, HD; pirfenidone, PFD; Ser, S; Thr, T. Data are presented as means ± SEM (A, B, and C) and were analyzed using one‐way ANOVA followed by a Bonferroni's multiple comparisons test. ^##^ *p <* 0.01, ^###^ *p <* 0.001, ^####^ *p <* 0.0001 compared with sham group; * *p <* 0.05, ** *p <* 0.01, *** *p <* 0.001, **** *p <* 0.0001 compared with UUO‐Veh or between groups under the line group; ns, not significant.

To test whether **DA** also targets β‐catenin signaling in vivo, we assessed the total and phosphorylated β‐catenin levels in the kidneys of UUO mice treated with vehicle or **DA**. Consistent with the results from cultured fibroblasts, **DA** reduced β‐catenin expression in UUO kidneys in a dose‐dependent manner (**Figure** **5**A). Meanwhile, the reduction of β‐catenin pro‐degradative phosphorylation at Ser33/37/45/Thr41 in UUO samples could be rescued by **DA** treatment, supporting the hypothesis that **DA** promotes β‐catenin ubiquitinated proteolysis in vivo (Figure 5B). Similar to cultured fibroblasts, **DA** failed to affect β‐catenin phosphorylation at Ser552/675 in UUO kidneys. Meanwhile, immunohistochemical staining revealed elevated β‐catenin primarily located in tubules and tubulointerstitium in fibrotic kidneys, which could be abolished by administration of **DA** (Figure 5C). As a result, a high dose of **DA** (20 mg kg^−1^) reverted UUO kidney close to that of healthy mice. To further characterize changes of β‐catenin in activated fibroblasts residing in tubulointerstitial compartment, we performed co‐immunofluorescence staining of α‐SMA and β‐catenin on kidney sections. More interestingly, elevated β‐catenin was detected in almost all α‐SMA positive myofibroblasts in UUO kidneys (Figure 5D). While, treatment of **DA** markedly reduced β‐catenin^+^ α‐SMA^+^ cell population in the tubulointerstitium, and high doses of the compound resulted in the near extinction of both stains.

### 2.6 **DA** Blocks the Pro‐Fibrotic β‐catenin Signaling by Inhibiting Cdc42 Activity

**Figure 6:**
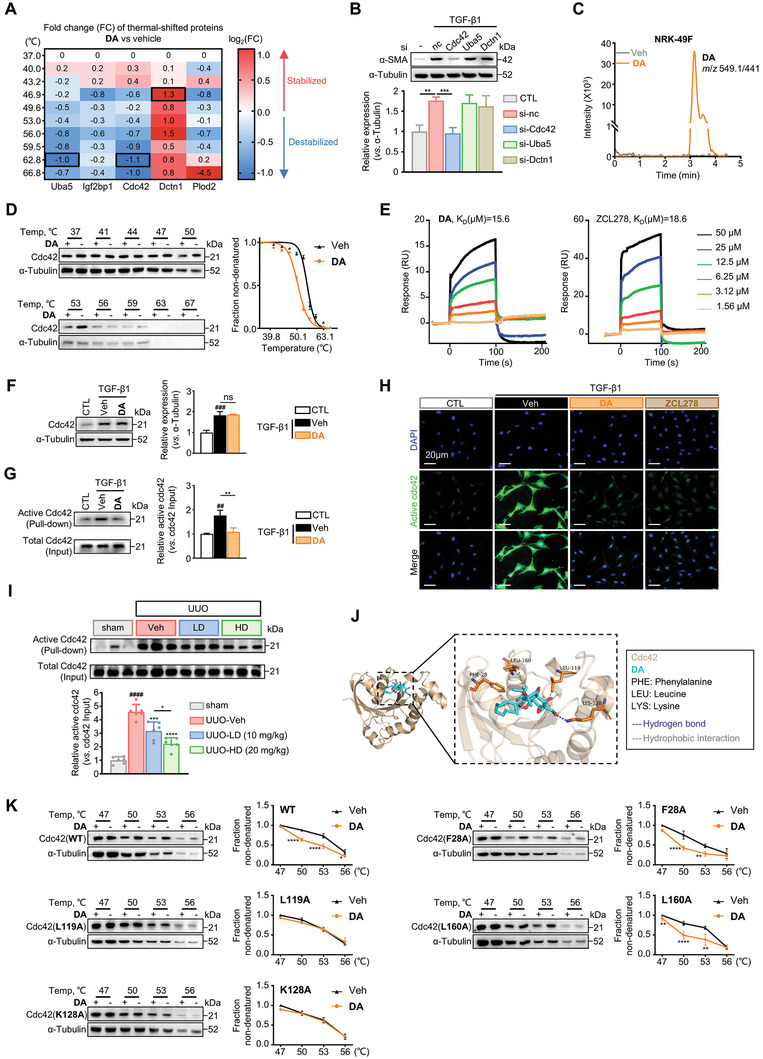
**DA** directly bound to Cdc42 and inhibited its activity. A) Heatmap indicating ‐log_2_(FC) of Δ*T*_m_ at each temperature for Uba5, Igf2bp1, Cdc42, Dctn1 and Plod2. Results are shown from 2 replicate experiments. B) Effects of the knockdown of Cdc42, Uba5, and Dctn1 on the protein levels of α‐SMA measured by western blotting (*n =* 3 per group). C) The HPCL/MS/MS analysis of NRK‐49F treated with vehicle or **DA**. D) CETSA analysis of intracellular binding between **DA** and Cdc42. Protein levels were investigated at different temperatures under the treatment of **DA** (5 µM) in NRK‐49F cells for 1 h. The graph shows the quantification of Cdc42 protein versus temperature points based on Western analyses (*n =* 3 per group). E) Surface plasmon resonance (SPR) analysis of interactions between **DA** or ZCL278 and Cdc42. F) Immunoblots of Cdc42 in NRK‐49F cells treated with vehicle or **DA** (*n =* 3 per group). G) Analysis of the active and total Cdc42 in NRK‐49F cells treated with vehicle or **DA** by pull‐down assay. Quantitative analysis of the grey value for active Cdc42/total Cdc42 ratio using ImageJ software (*n =* 3 per group). H) Representative immunofluorescence microscopy of active Cdc42. Serum‐starved NRK‐49F cells were treated with vehicle, 5 µM **DA**, or 5 µM ZCL278. Cells were probed with an active Cdc42 antibody (green). Nuclei were visualized by DAPI (blue). Scale bar, 20 µm. I) Analysis of the active and total Cdc42 in kidneys from mice treated with vehicle, low‐dose, or high‐dose **DA** by pull‐down assay (*n =* 6 per group). Results were quantified by ImageJ software. J) Molecular docking of Cdc42 and **DA**. K) CETSA analysis of intracellular binding between **DA** and wild‐type or mutant Cdc42 (*n =* 3 per group). Data were analyzed by two‐way ANOVA followed by Bonferroni's multiple comparisons test. Control, CTL; vehicle, Veh; WT, wild‐type; F, Phe; K, Lys; L, Leu. Data are presented as means ± SEM (B, D, F, G, I, and K) and were analyzed using one‐way ANOVA followed by a Bonferroni's multiple comparisons test unless otherwise stated. ^##^ *p <* 0.01, ^###^ *p <* 0.001, ^####^ *p* < 0.0001 compared with CTL or sham group; * *p <* 0.05, ** *p <* 0.01, *** *p <* 0.001, **** *p <* 0.0001 compared with Veh group or between groups indicated by a line; ns, not significant.

To further elucidate the pharmacological mechanism by which **DA** blocks the pro‐fibrotic β‐catenin signaling, we adopted the thermal proteome profiling (TPP) strategy that was established to dissect target proteins of small‐molecule compounds.^[^ ^35^ ^]^ We thus performed this approach in TGF‐β1‐activated NRK‐49F cells treated with/without **DA** (Figure S4A, Supporting Information). From 1350 protein candidates, 5 proteins (Uba5, Igf2bp1, Cdc42, Dctn1, and Plod2) were identified as the possible targets of **DA** according to the widely applied screening criteria^[^ ^35^, ^36^ ^]^ (Figure S4B; Table S2, Supporting Information). Among that, Plod2 was first excluded as it exhibited both thermodynamic stability and instability trends^[^ ^37^ ^]^ (**Figure** **6**A). After further applying a cutoff of ±1 for the maximum ‐log_2_ (fold change of *T*_m_),^[^ ^38^ ^]^ Uba5, Cdc42, and Dctn1 were identified as the most possible targets of **DA** and then underwent phenotypic screens. Among them, only the knockdown of *Cdc42* showed a dramatic inhibitory effect on α‐SMA, indicating that it would be the protein target of **DA** (Figure 6B; Figure S4C, Supporting Information). Cdc42 is synthesized as a cytosolic protein, but can also attach to the cytoplasmic face of cellular membranes by lipid anchors,^[^ ^39^ ^]^ which was true for the renal fibroblast we tested (Figure S5A, Supporting Information). Membrane Cdc42 primarily acts as a collection point and intersection for signal transduction.^[^ ^40^ ^]^ However, domains critical for Cdc42 activation and binding to effectors reside in the cytoplasm.^[^ ^41^ ^]^ Thus, we next sought to examine whether **DA** could enter into cells to interact with Cdc42. Detection of intracellular fractions by HPLC‐MS/MS analysis^[^ ^42^ ^]^ revealed the presence of significant amount of **DA** in **DA**‐incubated renal fibroblasts, indicating that **DA** can indeed be taken up by the cells (Figure 6C). Cellular thermal shift assay followed by western blot (CETSA‐WB) detection in fibroblasts showed that **DA** treatment could markedly induce thermal destabilization of Cdc42, suggesting the intracellular interaction of **DA** with Cdc42 (Figure 6D).

Subsequently, we performed the surface plasmon resonance (SPR) experiments to investigate the binding affinity between **DA** and Cdc42. We used ZCL278, a well‐known Cdc42 binding compound,^[^ ^43^ ^]^ as a positive control in SPR experiments. Interestingly, **DA** displayed a stronger binding affinity with Cdc42 than ZCL278, with a dissociation constant (*K*_D_) of 15.6 µM versus 18.6 µM, respectively (Figure 6E). However, **DA** did not regulate Cdc42 expression at both mRNA and protein levels (Figure S5B, Supporting Information; Figure 6F), which prompted us to investigate how **DA** affects Cdc42 activity. As a small G protein, the level of active Cdc42 can be indicated by the amount of GTP‐Cdc42.^[^ ^44^ ^]^ We detected GTP‐Cdc42 in renal fibroblasts by pull‐down assay and found that DA efficiently blocked TGF‐β1‐induced up‐regulation of GTP‐Cdc42, suggesting that the compound can reduce active Cdc42 (Figure 6G). Similar results were observed once detected with an antibody specific for immunofluorescence probing against active Cdc42 (GTP‐bound) (Figure 6H). We also found that Ang II or other stimuli could induce active Cdc42 in cells to varying degrees, while **DA** blocked these up‐regulation effects (Figure S5C, Supporting Information), and the same for TGF‐β1‐stimulated tubular epithelial cells (Figure S5D,E, Supporting Information). Importantly, we detected a marked increase in Cdc42 activity in UUO kidneys by pull‐down assay, while treatment with **DA** of either low‐dose or high‐dose significantly reduced active Cdc42 in fibrotic kidneys, and high‐dose **DA** exhibited a stronger effect (Figure 6I). Altogether, these findings suggest that **DA** serves as a potent Cdc42 inhibitor.

To further explore the exact binding sites of **DA** and Cdc42, we performed a molecular docking assay, and **DA** was predicted to form hydrogen bonds with Lys128 (K128) of Cdc42 as well as hydrophobic interactions with Phe28 (F28A), Leu119 (L119), and Leu160 (L160) (Figure 6J). Thus, four Cdc42 mutant‐expressing plasmids were constructed accordingly, and **DA**‐mutant Cdc42 interactions were examined by CETSA. As shown in Figure 6K, **DA** reduced the thermal stability of exogenous wild‐type (WT) Cdc42 as expected. However, this decrease was largely impaired by mutations in Leu119 (L119A) and Lys128 (K128A), but not by mutations in Phe28 (F28A) and Leu160 (L160A). Such results indicate that Leu119 and Lys128 might be responsible for the interaction between **DA** and Cdc42. Interestingly, Lys128 is located in the “insert domain (residues 122–135)” of Cdc42 which is essential for Cdc42 activation and binding to effectors.^[^ ^41^ ^]^ This suggests that **DA** may bind to Cdc42 via Leu119 and Lys128 to disturb its activity and interaction with the effectors.

### 2.7 **DA** Relies on Cdc42 to Regulate p‐PKCζ/p‐GSK‐3β/β‐catenin Axis and Exert Anti‐Fibrotic Activity

**Figure 7:**
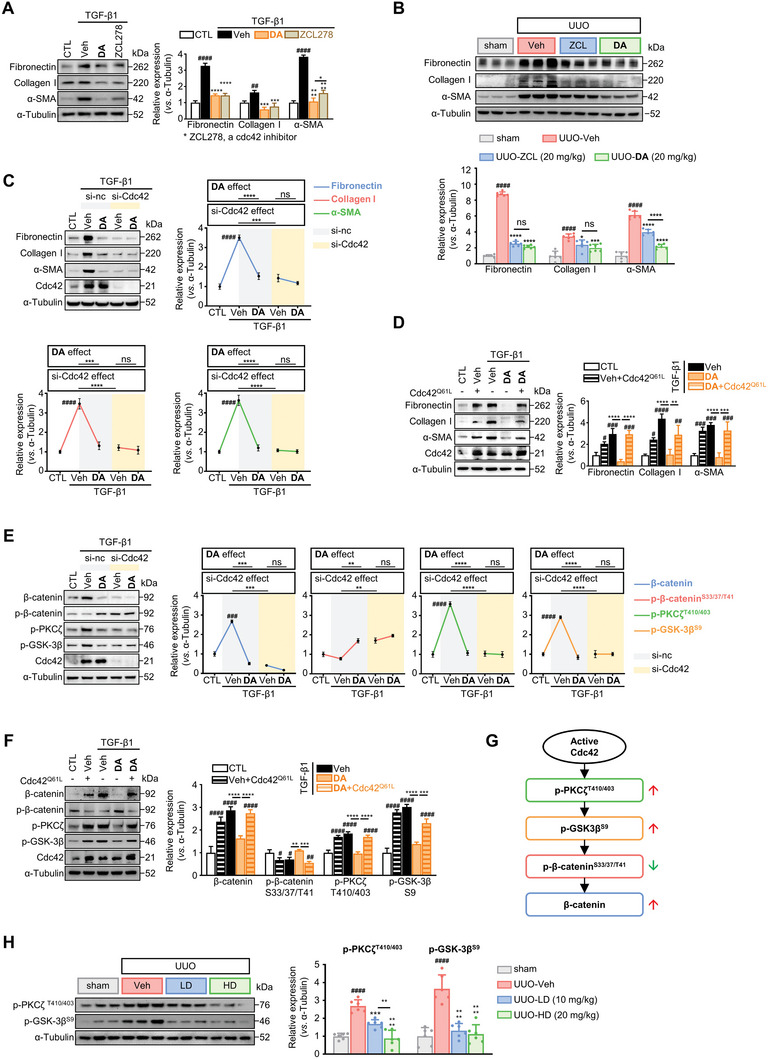
**DA** activated p‐PCKζ/p‐GSK‐3β‐mediated β‐catenin phosphorylation at S33/37/T41 by targeting Cdc42. A, B) Immunoblots showing the inhibitory effects of **DA** and ZCL278 on fibrotic markers in A) TGF‐β1‐activated NRK‐49F cells (*n =* 3 per group) and B) UUO mice (*n =* 6 per group). C, E) Influence of Cdc42 knockdown on (C) fibrosis‐related proteins and (E) p‐PKCζ/p‐GSK‐3β/p‐β‐catenin protein levels in NRK‐49F cells (*n =* 3 per group). NRK‐49F cells were transfected with si‐Cdc42 or negative control siRNA (si‐nc) and incubated with TGF‐β1+Veh or TGF‐β1+**DA** for C) 48 h or E) 12 h. D, F) Immunoblot analysis of D) fibrotic indicators and F) p‐PKCζ/p‐GSK‐3β/p‐β‐catenin axis in NRK‐49F cells transfected with constitutively active Cdc42 plasmid (Cdc42^Q61L^) (*n =* 3 per group). The post‐transfected NRK‐49F were treated with TGF‐β1+Veh or TGF‐β1+**DA** for D) 48 h or F) 12 h. G) Schematic diagram summarizing how active Cdc42 modulates p‐PKCζ/p‐GSK‐3β/p‐β‐catenin axis. H) Immunoblot analysis of p‐PKCζ and p‐GSK‐3β in mice kidneys of each group on the seventh days after sham or UUO surgery (*n =* 6 per group). Control, CTL; vehicle, Veh; NC, negative control; Ser, S; Thr, T. Data are presented as means ± SEM (A, B, C, D, E, F, and H) and were analyzed using one‐way ANOVA followed by a Bonferroni's multiple comparisons test. ^#^ *p <* 0.05, ^##^ *p <* 0.01, ^###^ *p <* 0.001, ^####^ *p <* 0.0001 compared with CTL group; * *p <* 0.05, ** *p <* 0.01, *** *p <* 0.001, **** *p <* 0.0001 compared with Veh group or between groups indicated by a line; ns, not significant.

It is essential to understand whether the target protein Cdc42 is sufficient to benefit the preventive role of **DA** against kidney fibrosis. To this end, a well‐known Cdc42 inhibitor, ZCL278, was used to validate its capability of inhibiting renal fibroblast activation in comparation with **DA**. As expected, 5 µM of ZCL278 markedly down‐regulated fibrosis‐associated proteins in renal fibroblasts, which displayed a similar effect as **DA** (**Figure** **7**A). ZCL278 at 20 mg kg^−1^ also significantly alleviated kidney fibrosis in UUO mice. It is worth emphasizing that **DA** at the same dose in vivo possessed a stronger inhibitory effect on α‐SMA than ZCL278, but similar effects on collagen I and fibronectin (Figure 7B). This was consistent with what we observed in vitro. However, ZCL278 treatment exhibited obvious systemic toxicity in mice, manifested by sustained weight loss and induction of liver dysfunction. In this comparison, **DA** was superior in safety profile (Figure S6A,B, Supporting Information).

To further determine whether the anti‐fibrotic effect of **DA** is Cdc42‐dependent, we depleted *Cdc42* in renal fibroblasts using small interfering RNA. Both **DA** and *Cdc42* knock‐down markedly reduced the expression of α‐SMA, fibronectin, and collagen I in renal fibroblasts. However, the inhibitory effects of **DA** on fibrotic markers were compromised upon *Cdc42* depletion, indicating that **DA** targets Cdc42 to perform its anti‐fibrotic effect (Figure 7C). Knockdown of *Cdc42* also significantly hindered the activation of fibroblasts by AngII and other stimuli (Figure S7, Supporting Information). Vice versa, transfection with a constitutively active Cdc42 (Cdc42^Q61L^)^[^ ^45^ ^]^ consistently promoted the activation of renal fibroblasts, with elevated ECM and α‐SMA levels (Figure 7D). Importantly, overexpression of active Cdc42 completely blocked the anti‐fibrotic potency of **DA**, indicating that the drug efficacy of **DA** depends on inactivating Cdc42.

Cdc42 deficiency is known to promote the degradation of β‐catenin, which depends on reduced phosphorylation of protein kinase C ζ (p‐PKCζ) and glycogen synthase kinase‐3β (p‐GSK‐3β).^[^ ^46^ ^]^ The decrease in p‐GSK‐3β actually corresponds to an increase in GSK‐3β, the latter primes β‐catenin proteolysis by phosphorylating it at Ser33/37/Thr41.^[^ ^47^ ^]^ We thus hypothesized that Cdc42 regulated the activation of renal fibroblasts via modulating p‐PKCζ/p‐GSK‐3β/β‐catenin axis. In line with this hypothesis, we found that TGF‐β1 up‐regulated p‐PKCζ (Thr410/403) and p‐GSK‐3β (Ser9) levels in cultured fibroblasts, companied by reduced p‐β‐catenin (Ser33/37/Thr41). However, **DA** and *Cdc42* knockdown decreased p‐PKCζ (Thr410/403) and p‐GSK‐3β (Ser9), but increased p‐β‐catenin (Ser33/37/Thr41), resulting in β‐catenin degradation (Figure 7E). As expected, knockdown of *Cdc42* again muted the regulatory activity of **DA** on the p‐PKCζ/p‐GSK‐3β/β‐catenin axis (Figure 7E). On the contrary, overexpression of active Cdc42 (Cdc42^Q61L^) initiated p‐PKCζ/p‐GSK‐3β signaling, suppressed p‐β‐catenin (Ser33/37/Thr41) and upregulated β‐catenin (Figure 7F). Transfection of Cdc42^Q61L^ also eliminated the effects of **DA** on regulating p‐PKCζ/p‐GSK‐3β/β‐catenin axis. All these observations from fibrotic protein expression, β‐catenin, and p‐PKCζ/p‐GSK‐3β signaling suggest that Cdc42 is essential in the regulation of this cascade (Figure 7G). These effects were further observed in UUO mice, where p‐PKCζ (Thr410/403) and p‐GSK‐3β (Ser9) increased in fibrotic kidneys and declined by **DA** administrations (Figure 7H). Together, these results support the hypothesis that **DA** exerts anti‐fibrotic activity by targeting Cdc42 activity to disrupt the p‐PKCζ/p‐GSK‐3β/β‐catenin axis.

### 2.8 Elevated Expression of Cdc42 Associates With Mouse and Human Kidney Fibrosis

**Figure 8:**
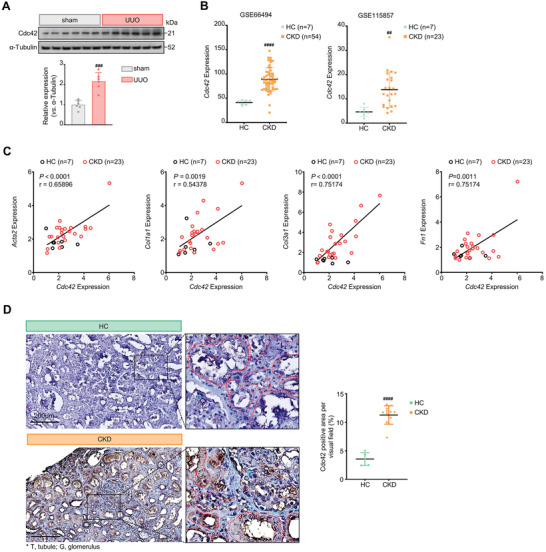
Higher expressions of Cdc42 were seen in both UUO mice and CKD patients. A) Immunoblot analysis of Cdc42 in mice kidneys of each group on the seventh day after sham or UUO surgery (*n =* 3 per group). B) Cdc42 transcript expression analysis for human kidneys from healthy controls (HC) and CKD patients in two public RNA‐sequencing datasets, GSE66494 and GSE115857. C) Correlation of the mRNA levels between Cdc42 and α‐SMA (*Acta2*), collagen I (*Col1a1*), collagen III (*Col3a1)*, and fibronectin (*Fn1*) were measured by Pearson's correlation coefficient (r) in GSE115857. The data is shown from 7 HC and 23 CKD patients. D) Immunohistochemical staining of Cdc42 in kidney sections from HC (*n* = 5) and CKD (*n* = 12). Scale bar = 200 µm. Blue dashed lines outline the glomerulus and red dashed lines for tubule. Data are presented as means ± SEM (A, B, and D) and were analyzed by students’ t‐test. ^###^ *p <* 0.001, ^####^ *p <* 0.0001 compared with sham or HC group.

The role of Cdc42 in kidney fibrosis has been rarely explored in vivo, and the exact mechanism linking Cdc42 to kidney fibrosis remains elusive. Increased expression of Cdc42 in TGF‐β1‐treated fibroblasts (Figure 6F) and its role in regulating fibrotic protein expression (Figure 7C,D) inspired us to investigate the relationship between Cdc42 expression and kidney fibrosis progression. To this end, we observed that fibrotic kidneys from UUO mice displayed a consistent and marked increase of Cdc42 compared with healthy kidneys (**Figure** **8**A). To disclose the clinical relevance of Cdc42 and kidney fibrosis in CKD patients, we queried the Gene Expression Omnibus of public gene expression profiles and found that CKD patients showed much higher Cdc42 transcript levels than healthy donors (Figure 8B). Moreover, Cdc42 was significantly positively correlated with the expression of fibrotic genes, including *Acta2*, *Col1a, Col3a1*, and *Fn1* (Figure 8C). To further characterize the expression pattern of Cdc42 in human fibrotic kidneys, we performed immunohistochemical staining on kidney specimens from CKD patients and healthy controls. In healthy kidneys, Cdc42 was slightly expressed in tubules and glomeruli, while scarcely detectable in tubulointerstitial compartment. In contrast, a significant increase of Cdc42 was detected in tubules, glomeruli, and interstitium in kidneys from CKD patients (Figure 8D), implicating the involvement of Cdc42 in kidney fibrosis. Taken together, these results suggest that high expression of Cdc42 is closely related to kidney fibrosis, thus pharmacological inhibition of Cdc42 may benefit patients with CKD.

## 3 Discussion

*W. chamaedaphne* is a medicinal plant documented in traditional Chinese medicine for the treatment of edema.^[^ ^48^ ^]^ However, its pharmacological activities and mechanism of action have not been fully explored. In the present study, the bioassay‐guided fractionation of 16 diterpenoids from *W. chamaedaphne* and subsequent assessment of their anti‐renal fibrotic effects led to the identification of **DA**. **DA** showed its activity in inhibiting renal fibroblast activation induced by various pro‐fibrotic or pro‐inflammatory cytokines. Essentially under these pathogenic stimuli, multiple intracellular signaling cascades can be triggered, converging on the PI3K/Akt pathway to activate Cdc42.^[^ ^49^ ^]^ And **DA**, as a Cdc42 inhibitor, terminates the transduction of these pro‐fibrotic signals. **DA** also exhibited a generalized inhibitory role in the transformation of various parenchymal cells into fibrotic phenotypes and their subsequent proliferation and migration, indicating a strong anti‐fibrotic efficacy. Such an outcome by **DA** is closely related to its targeting of Cdc42, blocking of downstream β‐catenin, and reduction of extracellular matrix components. Inactivation or knockdown of Cdc42 or the β‐catenin pathway has been reported to impair these cellular activities.^[^ ^50^ ^]^ In addition, the reduction of ECM components, which thereby greatly reduces growth factors and signaling molecules,^[^ ^51^ ^]^ is also one of the causes of this series of phenomena.

A recent study reported the isolation of daphnepedunin A (**DA**).^[^ ^52^ ^]^ In their study, **DA** displayed a therapeutic activity against HIV, but this activity was much weaker than the positive control gnidimacrin, and its underlying pharmacological mechanism remained untested. For inhibiting the fibrotic protein expression in activated renal fibroblasts and kidney fibrosis mouse models, **DA** exerted a beneficial effect, superior to that of PFD, a small molecule compound that has been approved to treat human idiopathic pulmonary fibrosis, and extensively explored in clinical trials for the treatment of kidney fibrosis.^[^ ^6a,b^ ^]^ Yet, the exact mechanism and drug targets of PFD are elusive to date, and the safety concerns still remain.^[^ ^5^ ^]^ Therefore, the high anti‐fibrotic activity and low systemic toxicity of **DA** underscored its translational importance.

In an attempt to unravel the signaling pathways affected by **DA**, we conducted bioinformatic analysis on RNA‐sequencing data followed by comprehensive experimental validation, including detailed detection of TGF‐β1 downstream effectors such as Smad2/3, to assure that the anti‐kidney fibrosis activity of **DA** attributed to its effects on the Wnt/β‐catenin pathway. The Wnt/β‐catenin cascade contributes to kidney development during embryogenesis and maintains tissue structure throughout human life.^[^ ^53^ ^]^ It is usually suppressed in adult kidneys but can be re‐activated and involved in tissue repair after kidney injury.^[^ ^29^ ^]^ Yet, persistent activation of the Wnt/β‐catenin cascade deepens crosstalk with other profibrotic signaling pathways and is sufficient to cause kidney fibrosis in CKD.^[^ ^30^, ^54^ ^]^ These adverse effects of Wnt/β‐catenin cascade may also involve its coupled renin‐angiotensin system (RAS), as well as other pathways that Wnt/β‐catenin signaling interacts with such as TGF‐β, Notch, and Hedgehog pathways, which are all implicated in fibrotic processes.^[^ ^55^ ^]^ Therefore, interfering with the Wnt/β‐catenin signaling can be an effective strategy to slow CKD progression. There is currently no drug to block the Wnt/β‐catenin pathway in clinical practice.^[^ ^56^ ^]^ In this context, the identification of **DA** as a novel inhibitor of the Wnt/β‐catenin pathway that controls β‐catenin degradation has significant translational interest. In particular, the suppressive action of **DA** on kidney fibrosis was no weaker than that of other Wnt/β‐catenin pathway inhibitors, including ICG001 and XAV939. ICG001 inhibits β‐catenin expression by binding to its transcriptional co‐factor CREB.^[^ ^57^ ^]^ This activity ICG001 on β‐catenin expression is indirect. The lack of CREB can be compensated by other β‐catenin co‐activators, which explains why ICG001 failed to reduce liver fibrosis in patients with cirrhosis.^[^ ^58^ ^]^ Direct effect on β‐catenin expression or metabolism may represent a better strategy for treating fibrosis, as in the case of XAV939. Similar to **DA**, XAV939 promotes β‐catenin degradation by stabilizing Axin, the scaffold protein of the β‐catenin “destruction complex”.^[^ ^34b^ ^]^ Nonetheless, in terms of anti‐fibrotic efficacy, **DA** seems much more effective than XAV939.

Understanding the pharmacological mechanisms of these anti‐fibrotic compounds can be challenging, which has been hindering their clinical application.^[^ ^59^ ^]^ TPP is an emerging technique that identifies ligand‐induced shifts of thermal stability in a high‐throughput manner, making it a great tool for tracking drug targets in living cells.^[^ ^35^ ^]^ Although most target proteins exhibit ligand‐induced stabilization, several studies have demonstrated that decreased thermal stability due to drug‐target binding also occurs.^[^ ^60^ ^]^ These new approaches have facilitated our identification of Cdc42 as a binding target of **DA**. Molecular docking and CETSA further revealed that **DA** interacts with Cdc42 through Lys128 and Leu119. Lys128 happens to be located in the Cdc42 cytoplasmic “insertion domain (residues 122–135)” where it plays a key role in Cdc42 activation and binding to effectors.^[^ ^41^ ^]^ Combined with our detection of **DA** peak inside the cells, we can speculate that **DA** entered renal fibroblasts and interacted with Cdc42 through Lys128 and Leu119 to inhibit its activity and the downstream GSK‐3β/β‐catenin signaling axis. Cdc42 regulates cell polarity, migration, and growth, and contributes to tumor metastasis and poor prognosis.^[^ ^61^ ^]^ During cardiac fibrosis following myocardial infarction, Cdc42 promotes cardiac fibroblast transdifferentiation into myofibroblasts.^[^ ^62^ ^]^ Cdc42 activation is important for renal EMT in vitro, although its exact role in tubulointerstitial fibrosis remains elusive.^[^ ^63^ ^]^ Here, we reported elevated Cdc42 levels in human CKD and mouse fibrotic kidney tissue. Experiments from Cdc42 knockdown by siRNA or inhibitor ZCL278 in vitro, as well as the overexpression of active Cdc42, proposed a hypothesis that Cdc42 is indispensable for fibroblast activation, pointing to a vital pathogenic role of Cdc42 in kidney fibrosis. On this basis, comparing **DA** with the known Cdc42 inhibitor ZCL278 could give us a clearer picture of the target intervention effect and drug properties. **DA** exhibited stronger anti‐fibrotic activity than ZCL278, likely due to the higher binding affinity of **DA** to Cdc42 than ZCL278. Besides, HPLC‐MS/MS showed that **DA** is mainly distributed in the kidney and liver in mice. Although it could be absorbed by the liver, no overt hepatotoxicity was detected, while ZCL278 at the same dose resulted in systemic and hepatic toxicity. Possibly of benefit, we observed that **DA** inhibited M2 polarization of macrophages and IFNg production in T cells by targeting Cdc42 (Figure S8, Supporting Information), which may facilitate its anti‐fibrotic activity in the kidney and deserves further study in the future.^[^ ^64^ ^]^

In conclusion, the anti‐renal fibrotic screen from natural diterpenoids extracted from *W. chamaedaphne* in the present study led to the discovery of **DA**, a novel antifibrotic agent, which is a promising lead deserving of further druggability assessment and applications. Its direct target protein Cdc42 is identified as a potential therapeutic target for the treatment of CKD.

## 4 Conclusion

**DA**, a natural small‐molecule diterpenoid isolated from the medicinal plant *W. chamaedaphne*, is identified to be a highly potent anti‐fibrotic agent, capable of blocking the activation of renal fibroblasts and alleviating kidney fibrosis in mouse models, being more effective than the positive control pirfenidone. Mechanistically, **DA** binds to Cdc42 and suppresses its activity, thereby reducing the downstream p‐PKCζ and p‐GSK‐3β levels, which leads to upregulated p‐β‐catenin (Ser33/37/Thr41) and the proteolysis of β‐catenin via the ubiquitin‐proteasome pathway.

## 5 Experimental Section

### Animal Experiments

Male C57BL/6J mice (GemPharmatech, Guangzhou, China) weighed 20–22 g were randomly assigned to three groups with 6 mice in each group as follows: 1) Sham‐operated mice with vehicle (Sham); 2) UUO mice with vehicle (Veh); 3) UUO mice treated with 150 mg kg^−1^ CE of *W. chamaedaphne* (CE); or five groups with 6 mice in each group as follows: 1) Sham‐operated mice with vehicle (Sham); 2) UUO mice with vehicle (UUO); 3) UUO mice treated with 10 mg kg^−1^ **DA** (UUO‐LD); 4) UUO mice treated with 20 mg kg^−1^ **DA** (UUO‐HD); 5) UUO mice treated with 250 mg kg^−1^ pirfenidone (UUO‐PFD); or four groups with 6 mice in each group as follows: 1) Sham‐operated mice with vehicle (Sham); 2) UUO mice with vehicle (UUO); 3) UUO mice treated with 20 mg kg^−1^ **DA** (UUO‐**DA**); 4) UUO mice treated with 20 mg kg^−1^ ZCL278 (UUO‐ZCL). To establish the UUO model, mice were given general anesthesia by intraperitoneal injection of pentobarbital (50 mg kg^−1^). The left ureter was exposed via a left flank incision, ligated with 4‐0 silk at two points, and cut between the 2 ligation points. The left ureter of sham‐operated mice was also exposed but without any ligation. For in vivo experiments, **DA**, pirfenidone, or CE was dissolved in DMSO and suspended in a hydrotropic agent containing 90% PBS and 10% cremophor EL (61791‐12‐6, Merck Millipore), which was then injected intraperitoneally for 7 consecutive days. Pirfenidone was used as positive control. The vehicle group received treatments of hydrotropic agent with 0.2% DMSO daily. After 7 days, mice were sacrificed and the left kidneys were harvested. All procedures were approved by the Animal Care and Use Committee of Sun Yat‐Sen University (Approved Number: 22020G).

### Plant Material and Extraction

Seeds of *W. chamaedaphne* Meisn. were collected in February 2020 in Shanxi Province, P. R. China, and were authenticated by professor Guihua Tang. A voucher specimen (accession number: HYH202002) was deposited at the School of Pharmaceutical Sciences, Sun Yat‐sen University. The dried buds of *W. chamaedaphne* (20 kg) were extracted with 95% EtOH (75 L × 3) at room temperature to give 3 kg of crude extract. The extract was suspended in H_2_O (3 L) and successively partitioned with petroleum ether, EtOAc, and n‐BuOH. EtOAc fraction that showed potent anti‐renal fibrosis activity was selected for further chemical investigation.

### Extraction of pMRF

For the extraction of pMRF: According to previous studies,^[^ ^65^ ^]^ C57BL/6J mice were sacrificed and kidneys were collected. The renal capsules were carefully peeled back and small sections of cortex were cut off. Then the renal tissues were cut into pieces in the 1.5 mL Eppendorf tubes and transferred to a 25T culture flask. Tissue explants in 1 mL DMEM/F12 (20% FBS) were incubated overnight before an additional 2 mL DMEM/F12 (20% FBS) was supplemented. After 72 h, the initial cell populations were established and cellular debris was removed. The culture medium was changed twice weekly for 10 days until the cell monolayer reached ≈75%. The extracted cells were defined as fibroblasts based on positive staining for vimentin, α‐SMA, and collagen I, together with negative staining for E‐cadherin (data not shown).

### Cell Culture and Treatment

The following cell lines were utilized in this study: normal rat kidney fibroblasts (NRK‐49F) (CRL‐1570, ATCC), tubular epithelial cells (NRK‐52E) (CRL‐1571, ATCC), and pMRF, all of which were maintained DMEM/F12 (C11330500BT, Gibco) supplemented with 10% fetal bovine serum (FBS) (10099141, Gibco), 100 U mL^−1^ penicillin and 100 µg mL^−1^ streptomycin (15140122, Gibco). RAW 264.7 (TIB‐71, ATCC) and LX2 (SCC064, Sigma) were maintained in DMEM (C11965500BT, Gibco) supplemented with 10% (FBS). And JURKAT cell (TIB‐152, ATCC) was cultured in 1640 (C11875500BT, Gibco) supplemented with 10% FBS. Cells were seeded in six‐well plate (1 × 10^5^ cells per well) containing 10% FBS until it reached ≈70% confluence for in vitro experiments. Cells were starved in serum‐free medium for 24 h and then were treated with 10 ng mL^−1^ (NRK‐49F and NRK‐52E) or 5 ng mL^−1^ (LX2) recombinant TGF‐β1 (240‐B‐002, R&D Systems), with or without small‐molecule compound or CE as instructed concentration for 6, 12, 24, or 48 h. RAW 264.7 was stimulated by 20 ng mL^−1^ IL‐4 (404‐ML‐010, R&D Systems). JURKAT was activated by 25 µL mL^−1^ anti‐CD3&CD28 (10971, Stemcell). The 16 diterpenoids used in cellular experiments were dissolved in DMSO (D2650, Sigma) and added to cell cultures at 10 µM concentration. CE was also dissolved in DMSO and added to cells at instructed concentrations. Angiotensin II (A1042, APExBio, 10 µM), IL‐17A (7956‐ML‐025, R&D, 50 ng mL^−1^), LPS (L2630‐10MG, Sigma, 25 ng mL^−1^), IL‐1β (401‐ML‐005, R&D, 10 ng mL^−1^), ICG001 (HY‐14428, Med Express, 5 µM), XAV939 (HY‐15147, Med Express, 5 µM), enalapril (HY‐B0331, Med Express, 5 µM) and valsartan (HY‐18204, Med Express, 5 µM), pirfenidone (P1871, Tokyo chemical industry, 5 µM), BTZM (HY‐10227, Med Express, 1 nM) or ZCL278 (HY‐13963, Med Express, 1 nM) were added to cell cultures in the indicated experiments. In the vehicle group, cells were treated with 0.1% DMSO.

### Western Blot Analysis

Total protein in NRK‐49F, NRK‐52E, LX2 cells, and renal tissue were extracted in RIPA buffer (20‐188, Merck Millipore). The concentration of total protein was measured according to the BCA kit instructions (23227, Thermo Fisher Scientific). Cytoplasmic and nuclear proteins were extracted and separated according to the instructions of the NE‐PER nuclear and cytoplasmic extraction kit (78833, Pierce). Membrane and cytoplasmic proteins were extracted and separated using the Membrane and Cytosol Protein Extraction Kit (P0033, Beyotime). Protein (30 µg) was separated on 10% SDS‐PAGE (ET15010LGel, ACE) and transferred to 0.45 µm PVDF membranes (IPVH00010, Merck Millipore). The membranes were blocked in 5% skim milk (232100, BD Biosciences) for 1 h at room temperature. Then, the membranes were incubated with a primary antibody overnight at 4 °C and an HRP‐conjugated secondary for 1 h at room temperature. The signals were detected using Immobilon Western HRP Substrate (WBKLS0500, Merck Millipore). Antibodies used in the experiments are listed in Table S3 (Supporting Information). The signals were quantified by measuring the intensity using ImageJ and normalizing them to the signal for the α‐Tubulin.

### RT‐qPCR

Total RNA was extracted from mice kidney and NRK‐49F subjected to RNeasy Mini Kit (74106, QIAGEN), and reverse transcription was performed from 1 µg of total RNA to generated cDNA templated using HiScript II Q RT SuperMix for qPCR kit (R223‐01, Vazyme). Quantitative RT‐PCR was performed using LightCycler 480 SYBR Green I Master (4887352001‐1, Roche) and sequence‐specific primers. Gene expression values were calculated using the 2 − ΔΔCT method and normalized to β‐actin. The primers used are listed in Table S4 (Supporting Information).

### Immunofluorescence Staining

NRK‐49F cells were seeded (1 × 10^4^ cells mL^−1^) into 24‐well plates containing rounded glass cover slips. Cells were incubated with or without TGF‐β1 (10 ng mL^−1^), followed by the addition of 5 µM **DA** or vehicle (DMSO). Following 12 or 48 h of treatment, cells were washed and fixed with ice‐cold 4% paraformaldehyde (JTW003, Tongmen technology). Cells were then permeabilized with 0.2% Triton X‐100 (T8200, Solarbio), and blocked with 5% donkey serum (D9663, Sigma) for 1 h at room temperature, followed by incubation with primary antibodies: anti‐α‐SMA‐Cy3 (Sigma C6198, 1:2000), anti‐β‐catenin (CST 8480, 1:100) or anti‐active Cdc42 (NewEast Biosciences 26905, 1:100) overnight at 4 °C. Coverslips were then incubated with AlexaFlour 488‐conjugated donkey anti‐rabbit secondary antibody (Invitrogen A21206, 1:1000) for 1 h at room temperature. The cells were counterstained with DAPI (Invitrogen D1306, 1:1000) to visualize the nuclei. Images were taken by laser scanning confocal microscopy (Zeiss, Jena, Germany).

As for mouse renal tissue sections, the kidneys were fixed in 4% paraformaldehyde overnight and then dehydrated in 30% sucrose for 8 h at 4 °C. Kidneys were then embedded in paraffin and cut into 6‐µm‐thick sections. The sections were permeabilized and blocked as demonstrated above, followed by incubation with anti‐β‐catenin and anti‐α‐SMA‐Cy3 antibodies overnight at 4 °C. The slides were stained with fluorescence‐labeled secondary antibodies for 1 h at room temperature. Nuclei were stained with DAPI. The slides were observed using the Zeiss microscope.

### Cell Proliferation Assay

The MTS assays were performed using a CellTiter 96 Aqueous One‐Solution Cell Proliferation Assay kit (G3582, Promega). Cells (5 × 10^3^ cells per well) were seeded in 96‐well plates treated with 5 µM **DA** for the indicated time. 200 µL of MTS reagent was added to each well, and after 90 min, the absorbance of the samples was read at 490 nm. EdU cell proliferation staining was performed using an EdU kit (C0071, Beyotime). Briefly, cells (1 × 10^4^ cells per well) were seeded in 24‐well plates for 48 h and subsequently incubated with EdU solution for 2 h. Then cells were directly fixed with 4% paraformaldehyde for 15 min to be observed using confocal microscopy, or trypsinized before being fixed for flow cytometry. Fixed cells were permeabilized with 0.1% Triton X‐100 for another 15 min. The cells were incubated with the Click Reaction Mixture (containing 430 µL Click Reaction Buffer, 20 µL CuSO_4_, 1 µL Azide 488 and 50 µL Click Additive solution per 1 mL) for 30 min at room temperature in a dark place and then incubated with DAPI for 10 min.

### Wound healing assay

Cells were seeded into six‐well plates in a complete medium until they created a confluent monolayer. Then, cells were cultured in a medium containing low FBS (0.5%) and the monolayer was scraped in a straight line with a P200 pipette tip to create a “scratch wound”, followed by washing with PBS and incubation with complete culture medium with or without TGF‐β1 and 5 µM **DA**. Images of the wounded cell monolayers were taken under microscopy (Olympus, Tokyo, Japan) at 0, 24, and 48 h after wounding. The cell healing rate was quantified using measurements of the area covered by migrating cells after culture. The area covered with cells was measured using the “measurement” function in Image J Software.

### Transwell Assay

Cell migration was measured using 24‐well transwell inserts (8 µm pore size) (3422, corning). pMRFs were seeded (5 × 10^5^ cells per well) into the upper chamber in an FBS‐free medium, while the lower chamber contained a complete medium supplemented with 10% FBS. TGF‐β1 (10 ng mL^−1^) with vehicle, or TGF‐β1 with **DA** (5 µM) was added and incubated for 24 h. Then, the medium was removed and cells in the lower chamber were stained (crystal violet) and observed with a Nikon microscope.

### RNA‐seq

Total RNA was isolated from TGF‐β1‐treated NRK‐49F cells with or without the incubation of **DA** for 48 h using Trizol (15596026, Invitrogen) according to the manufacturer's protocol. RNA quality was assessed on an Agilent 2100 Bioanalyzer (Agilent Technologies, CA, USA) and checked using RNase‐free agarose gel electrophoresis. After total RNA was extracted, eukaryotic mRNA was enriched by Oligo(dT) beads. Then the enriched mRNA was fragmented into short fragments using fragmentation buffer and reversely transcribed into cDNA using NEBNext Ultra RNA Library Prep Kit for Illumina (New England Biolabs, USA, NEB #7530). The purified double‐stranded cDNA fragments were end‐repaired, a base added, and ligated to Illumina sequencing adapters. The ligation reaction was purified with the AMPure XP Beads (1.0X). Ligated fragments were subjected to size selection by agarose gel electrophoresis and polymerase chain reaction (PCR) amplified. The resulting cDNA library was sequenced using Illumina Novaseq6000 by Gene Denovo Biotechnology Co. (Guangzhou, China). The mapped reads of each sample were assembled by using StringTie v1.3.1 in a reference‐based approach. For each transcription region, an FPKM (fragment per kilobase of transcript per million mapped reads) value was calculated to quantify its expression abundance and variations, using RSEM software. RNAs differential expression analysis was performed by DESeq2 software between two different groups (and by edgeR between two samples). The genes/transcripts with the parameter of false discovery rate (FDR) below 0.05 and absolute fold change ≥ 2 were considered differentially expressed genes/transcripts.

### In Vitro Ubiquitylation Assay

NRK‐49F cells were co‐transfected with HA‐Ub and Flag‐β‐catenin, along with a mock vector in the presence of BTZM (1 nM). Cells were lysed in NP‐40 buffer (P0013, Beyotime) and vortexed. The lysates were incubated with protein A/G magnetic beads (HY‐K0202, MCE) and tubes were rotated at 4 °C for 4 h to exclude the non‐specific binding between beads and cell lysates. Beads were then spun down and precleared lysates were incubated with anti‐Flag antibody (Proteintech 20543‐1‐AP, 1:30) at 4 °C for 4 h. Fresh beads were added to the IP samples, and the samples were rotated at 4 °C overnight. Subsequently, beads were spun down and washed twice with PBS containing 0.05% Tween20 (8.22184.0500, Merckmillipore). The beads were resuspended with SDS loading dye (LT101, EpiZyme) and boiled. The pull‐down proteins were resolved by SDS‐PAGE for immunoblotting using an anti‐HA antibody (Santa Cruz sc‐7392, 1:100).

### TPP

TPP was performed according to previous reports^[^ ^35^, ^36^ ^]^ as follows:.

### Cell Lysis Preparation

For cellular thermal shift assay, NRK‐49F cells with 70 to 80% confluence in a 15 cm culture dish were incubated with TGF‐β1 with simultaneous treatment of **DA** or vehicle (DMSO) for 3 h. Cells were harvested and washed once with PBS, then suspended in 1 mL of PBS supplemented with proteinase and phosphatase inhibitors (Roche). The cell suspension was evenly distributed into ten 0.2 mL PCR tubes with 100 µL volume by cell counting and each tube was designated a temperature point. Samples were heated at their designated temperatures for 2 min in thermal cycler. Immediately after heating, tubes were removed and incubated at room temperature for 3 min. After that, tubes were immediately snap‐frozen in liquid nitrogen. In order to lyse the cells, three freeze and thaw cycles in liquid nitrogen were performed. The tubes were vortexed briefly after each thawing. Cell lysates were collected by centrifuging samples at 13 000 g for 15 min at 4 °C. Next, cell lysates were subject to protein precipitation with acetone and then reconstituted in 50 µL of 8 M Urea in 100 mM triethylammonium bicarbonate (TEAB). Samples were next subjected to reduction of Cys‐Cys bonds with 20 mM dithiothreitol (DTT), and alkylation with 40 mM iodoacetamide (IAA) to protect the reduced Cys residues. Samples were diluted to have 0.7 m urea and were digested in‐solution overnight using 0.5 µg µL^−1^ trypsin (ThermoFisher Scientific) to derive peptides.

### Peptide Enrichment

The peptides were “de‐salted” using Sep‐Pak Vac 1cc C18 Cartridges (WAT054955, Waters). Briefly, columns were first washed sequentially with 1) Methanol (0.1% trifluoroacetic acid (TFA), 1 mL)‐three times, 2) Methanol/H_2_O 50/50 (v/v; 0.1% TFA; 1 mL)‐three times, and 3) MS‐grade water (0.1% TFA, 1 mL)‐three times. Peptides from each “digestion” solution were then subjected to immobilization on C18 material. Then, the peptide‐bound C18 columns were washed with MS‐grade water (0.1% TFA, 1 mL)‐two times, MS‐grade water (0.1% FA, 1 mL)‐one time and then eluted with 500 µL of acetonitrile (ACN) /H_2_O 70/30 (v/v; 0.1% FA) two times. All elution fractions were collected and subjected to complete dryness using a speed vacuum system.

### Tandem Mass Tags (TMT) Labeling

The dried samples were then respectively subjected to TMT‐based labeling using tenplex kits (TMT10plex Isobaric Label Reagent Set, ThermoFisher Scientific). The TMT channels—TMT126, TMT127N, TMT127C, TMT128N, TMT128C, TMT129N, TMT129C, TMT130N, TMT130C, and TMT131 were employed to label peptide solutions derived from the 37.0, 40.0, 43.2, 46.9, 49.6, 53.0, 56.0, 59.5, 62.8, and 66.8 °C temperature treatments. Each dried sample was reconstituted in 100 µL of 50 mM TEAB and the labeling reagents were dissolved in 40 µL of (ACN) Reconstituted peptide solutions were mixed with 40 µL labeling reagent solution and then shaken at room temperature for 4 h to label the peptides. Next, 8 µL of 5% hydroxylamine was added to stop the reaction. Labeled peptide solutions were mixed together and subjected to complete dryness in a speed vacuum system. The labeled peptides were re‐constituted in 0.1% formic acid (50 µL) prior to nano‐LC‐MS/MS analysis.

### Nano‐LC‐MS/MS Analysis

Samples were analyzed using Nano LC‐Q Exactive Plus (Thermo Fisher Scientific,). Buffer A consisted of 0.1% formic acid in water and buffer B contained 0.1% (vol/vol) formic acid in 80% (vol/vol) MS‐grade ACN in water. Nano‐LC was operated in a single analytical column setup using PicoFrit Emitters (75 µm inner diameter, New Objectives) packed in‐house with Reprosil‐Pure‐AQ C18 phase (1.9 µm particle size, 16 cm column length) at a flow rate of 200 nL min^−1^. All samples were separated using a 120 min gradient (3 to 32% Buffer B in 95 min, from 32 to 100% in 10 min, followed by isocratic elution at 100% for 15 min). The nanospray ion source was operated at a 2.2 kV spray voltage and 275 °C heated capillary temperature. The mass spectrometer was set to acquire full scan MS spectra (355–1700 m/z) for a maximum injection time of 100 ms at a mass resolution of 70 000 and automated gain control (AGC) target value of 5 × 10^5^. The ddMS2 IT higher‐energy collision dissociation (HCD) scan used a quadrupole isolation window of 1.6 m/z and auto m/z scan range mode with first m/z of 120. The mass resolution was 35 000, the maximum injection time was 75 ms, and the AGC target value was 5 × 10^4^. The dynamic exclusion was set to 60 s at an exclusion window of 10 ppm with a cycle time of 3 s for all methods.

### Mass Spectrometry Data Processing

The datasets were processed with raw files being created by Thermo Proteome Discoverer 2.2.0 software for peptide and protein identification. Data were searched against the Rattus norvegicus reference proteome, with common contaminants and serum proteins included in FASTA format. The following settings were used: the trypsin cleavage rules with a maximum of 2 missed cleavages, the precursor mass tolerance was 10 ppm, and the fragment mass tolerance was 0.02 Da. The remaining parameters were set to default according to the developer's instructions.

Data processing was based on previous reports.^[^ ^35^, ^36^ ^]^ Briefly, a flat slope of the melting curve relates to lower *T*_m_ reproducibility, thus proteins with an absolute slope below 0.06 were excluded and plotted in gray. In addition, proteins with an absolute slope above 0.06 were plotted in blue and their *T*_m_ shifts were further evaluated. Criteria for a significant *T*_m_ shift were listed as follows: 1) melting point shifts in **DA** versus DMSO experiments had the same sign; 2) both the melting point differences (Δ*T*_m_) in the **DA** versus DMSO experiments were greater than the Δ*T*_m_ between the two DMSO experiments. **DA**‐induced *T*_m_ shifts that passed the significance criteria were shown in red.

### HPLC‐MS/MS Analysis

NRK‐49F cells were treated with 5 µM **DA** or vehicle for 48 h and then harvested with PBS by several washings to remove **DA** outside the cells. Cells were lysed with 50% (v/v) water/methanol followed by several shock freeze‐thaw cycles and supernatants were collected for analysis. For tissues, heart, liver, spleen, lung, and kidney from mice injected with 20 mg kg^−1^ **DA** or vehicle were collected after washing with saline. The homogenate was centrifuged at 10 000 g for 10 min at 4 °C and tissue supernatants were subjected to HPLC‐MS/MS. Analysis was performed on a HPLC system of Thermo Vanquish Core (Thermo Scientific), which was coupled to a Thermo TSQ Quantum Access MAX instrument (Thermo Scientific) operating in positive ion electrospray ionization (ESI) and multiple reaction monitoring (MRM) scan type. Water (containing 0.1% formic acid, mobile phase A) and methanol (mobile phase B) were used as the mobile phases. A T3 C18 Column (2.1 × 100 mm, 3 µm, Waters) was used for analyte separation. The column oven temperature was set to 40 °C. All samples (10 µL) were separated using isocratic elution (20% Buffer A and 80% Buffer B). The system flow rate was 0.25 mL min^−1^. The quantification was performed using transitions of *m*/*z* 549.1 > 441, with the following parameters: spray voltage, 3.2 kV; capillary temperature, 250 °C; sheath gas pressure, 45 psi; auxiliary gas pressure, 10 psi; Ion transfer tube temperature, 350 °C; collision energy, 19 eV; RF Lens, 79 V.

### CETSA‐WB

NRK‐49F cells (1 × 10^6^) were plated in a 15 cm dish and incubated with DMSO or **DA** for 4 h after attachment. Cells were trypsinized and washed with PBS. Cells were divided into ten groups applied to different temperatures including 37, 41, 44, 47, 50, 53, 56, 59, 63, and 67 °C respectively for 2 min and then incubated at 25 °C for 3 min. Cellular protein was extracted with RIPA buffer and the protein bands were detected with western‐blotting using anti‐Cdc42 or anti‐His antibodies.

### Surface Plasmon Resonance

SPR was determined using a Biacore 8K instrument (GE Healthcare, Uppsala, Sweden). The recombinant Cdc42 protein (230‐00660‐100, RayBio) was immobilized on a sensor chip (CM5) using the amine‐coupling method according to standard protocols. Cdc42 protein was diluted in sodium acetate buffer, pH 4.5. Various concentrations of **DA** and ZCL278 were subsequently injected as analytes. To estimate the affinity, the binding assay was examined at 25 °C at a flow rate of 30 µL min^−1^ using PBS buffer. The affinity constants of binding were obtained using the 1:1 Langmuir binding model via BIA evaluation software.

### Cdc42 Activation Assay

The assay was performed according to the instructions of Cdc42 activation assay biochem kit (BK034, Cytoskeleton) according to the instructions. NRK‐49F cells were treated with 10 ng mL^−1^ TGF‐β1 or TGF‐β1 together with 5 µM **DA** for 30 min. Untreated NRK‐49F cells were used as controls. Protein was extracted from the cell lysates and 20 µL of lysates were aliquoted for the quantification of total Cdc42. The remaining lysate was frozen in liquid nitrogen. The total Cdc42 was quantified by western blot in each sample. According to the blot result, the concentration of total Cdc42 was equalized. PAK‐PBD beads (200 µL) were incubated with the samples at 4 °C on the rotator for 1 h. Subsequently, beads were pelleted by centrifugation at 4000 g at 4 °C for 1 min, and the supernatant was carefully removed. Beads were washed and 10 µL loading buffer was added to resuspend the beads. Samples were boiled for 2 min and the levels of GTP‐Cdc42 were analyzed by western blot.

### Molecular Docking

The crystal structure of Cdc42 used for docking was downloaded from the PDB database with PDB ID 4js0. The 3D structure of the small molecule was constructed from Chem3D and energy minimization was carried out under the MMFF94 force field. AutoDock Vina 1.1.21 software was adopted for molecular docking and PyMol 2.5.42 was used to remove water molecules, salt ions, and small molecules from the protein. The docking box was then set up to encase the entire protein structure. In addition, all processed small molecules and receptor proteins were converted into the PDBQT format required for docking by ADFRsuite 1.03. For docking, the exhaustiveness of the global search was set to 32, and the rest of the parameters were kept at default. The output docking conformation with the highest scoring was considered by us as the bound conformation, and finally, PyMol 2.5.4 was used for visualization.

### SiRNA and Plasmids Transfection

Cdc42, Uba5, and Dctn1 siRNA or negative control (Hanyi, Guangzhou, China) was transfected into NRK‐49F cells via Lipofectamine 3000 reagent (L3000015, Invitrogen). Briefly, NRK‐49F cells were plated into the six‐well plate in a complete DMEM/F12 medium without antibiotics. After cells reached 50% confluence, the initial medium was replaced by Opti‐MEM medium (31985070, Gibco) containing 120 nM Cdc42‐siRNA and 5 µL Lipofectamine 3000 reagent. Following incubation for 8 h, the cells were transferred to a complete DMEM/F12 medium and incubated until they reached 80% confluence. Subsequently, TGF‐β1 and **DA** were added into the medium. After 24 or 48 h, cells were harvested for western blot. RAW264.7 and Juarkat were also transfected with si‐Cdc42 using the same procedure. These sense sequences are listed in Table S5 (Supporting Information).

NRK‐49F cells were seeded in a 10 cm culture dish (1 × 10^6^ cells per dish) containing 10% FBS until they reached ≈80% confluence. Fresh culture medium was added to cells 2 h before the transfection. 10 µL NEOFECT DNA transfection reagent and 10 µg plasmid (HA‐Ub, Flag‐β‐catenin, Cdc42^Q61L^, His‐Cdc42^WT^, His‐Cdc42^F28A^, His‐Cdc42^L119A^, His‐Cdc42^K128A^, or His‐Cdc42^L160A^) were incubated for 10 h. Afterward, cells were exposed to different treatments indicated in corresponding experiments.

### Serum Biochemical Testing

Blood samples from anesthetized mice were collected in 1.5 mL EP tubes by cardiac puncture and the sample tubes were left in a standing position at room temperature for 1 h. Then the blood samples were centrifuged at 3000 rpm for 10 min and the upper layer serum was collected. AST and ALT were measured with an automatic biochemical detector (Roche, Basel, Switzerland).

### Picrosirius Red Staining

Seven‐day obstructed kidney tissues were fixed in 4% formalin and embedded in paraffin and then cut into 6 µm sections and stained with Picro Sirius Red Stain Kit (ab150681, Abcam) according to the instructions. Briefly, the sectioned slides were incubated with Picro Sirius Red Solution for 1 h at room temperature. Slides were then rinsed with fresh 0.5% acetic acid solution twice and dehydrated with 100% ethanol three times. Tissue slides were cleared with xylene for 10 min and mounted with synthetic resin mounting medium (99302, sh‐bbmx).

### Immunohistochemical Staining

Immunohistochemical analysis was performed with 6 µm‐thick mouse or human kidney sections that had been de‐waxed with xylene and hydrated using sequential ethanol volumes (100%, 95%, 90%, 80%, 75%) and distilled water. Endogenous peroxidase was blocked with 3% hydrogen peroxide for 15 min. Antigen retrieval was performed by heating sections in sodium citrate buffer (pH 6.0) (PN4115, G‐CLONE). The sections were then incubated with 10% donkey serum (D9663, Sigma) at room temperature for 1 h, followed by incubation with the β‐catenin antibody (1:100) or Cdc42 antibody (1:50) at 4 °C overnight. Slides were subsequently washed with PBS for four times, incubated with a secondary antibody conjugated with HRP (1:1000) at room temperature for 1 h, and washed with PBS, followed by incubation with 3,3′‐diaminobenzidine (DAB) solution (K5007, Dako). The slides were counterstained with hematoxylin and mounted with synthetic resin. Signals of β‐catenin or Cdc42 were detected by microscopy (Olympus, Tokyo, Japan). All samples were semi‐quantitatively assessed using ImageJ.

### Patient Specimens

Human kidney specimens were obtained from CKD patients undergoing renal biopsies in the First Affiliated Hospital of Sun Yat‐sen University. The clinical characteristics of patients are provided in Table S6 (Supporting Information). Normal renal specimens of non‐tumor renal tissues from patients with renal cell carcinoma were used as controls. Informed consent was obtained from all individual participants included in the study and recorded in the electronic database. The research was conducted following the guidelines set by the medical ethical committees of the First Affiliated Hospital of Sun Yat‐sen University (Approved Number: [2016] 215).

### Statistical Analyses

All data were presented as mean ± SEM unless stated otherwise. The sample sizes (*n*) and probability (*p*) values for each experiment were indicated in the figure legends. Data were analyzed and plotted using Prism (GraphPad Software). The difference between the two groups was examined by student's t‐test. Statistical significance of more than two groups was assessed by the analysis of variance (ANOVA), followed by a Bonferroni multiple comparisons test. *p*‐Values <0.05 were considered significant. The correlation between the two variables was measured by Pearson's correlation coefficient (r). CETSA and bodyweight results were analyzed by two‐way ANOVA, followed by the Bonferroni multiple comparisons test.

## Conflict of Interest

The authors declare no conflict of interest.

## Author Contributions

X.H. and L.G. contributed equally to this work. X.R.H. conceived of and designed experiments. X.R.H. and L.G. wrote the manuscript with input from all authors. Z.W.T. performed si‐RNA experiments and R.N.L. helped with renal section staining. Z.L., F.L., and C.J.Z. processed and analyzed the RNA‐sequencing data. X.H., R.L.Z., and J.N.S helped to establish the animal model. J.Y. performed EdU experiments. N.L. measured serum AST and ALT. W.X.P., X.Y.L., J.Q.T., and J.J.F. helped with the western blotting. Q.W., X.W., J.B.L., and X.H.Z. constructed the plasmids used in this study. W.C. Q.H.L. and H.P.M. provided human renal tissues. Y.Z., Y.S., G.P.S., and J.P.G revised and finalized the manuscript. All authors contributed to the article and approved the submitted version.

## Supporting information

Supporting Information

## Data Availability

The data that support the findings of this study are available in the supplementary material of this article.
