# Supplementary material for: A Natural Small Molecule Mitigates Kidney Fibrosis by Targeting Cdc42‐mediated GSK‐3β/β‐catenin Signaling
Source: Adv Sci (Weinh). 2024 Jan 19;11(13):2307850. doi: 10.1002/advs.202307850 (PMC10987128; doi:10.1002/advs.202307850)
Supplement: Supplementary file 1 — Supporting Information [file ADVS-11-2307850-s001.pdf]

ADVANCED SCIENCE
Open Access

Supporting Information

for *Adv. Sci.*, DOI 10.1002/advs.202307850

A Natural Small Molecule Mitigates Kidney Fibrosis by Targeting Cdc42-mediated GSK-3β/β-catenin Signaling

Xinrong Hu, Lu Gan, Ziwen Tang, Ruoni Lin, Zhou Liang, Feng Li, Changjian Zhu, Xu Han, Ruilin Zheng, Jiani Shen, Jing Yu, Ning Luo, Wenxing Peng, Jiaqing Tan, Xiaoyan Li, Jinjin Fan, Qiong Wen, Xin Wang, Jianbo Li, Xunhua Zheng, Qinghua Liu, Jianping Guo, Guo-Ping Shi, Haiping Mao, Wei Chen, Sheng Yin and Yi Zhou**

Supporting Information

A Natural Small Molecule Mitigates Kidney Fibrosis by Targeting Cdc42-mediated GSK-3β/β-catenin signaling

Xinrong Hu[†]*, Lu Gan*[†]*, Ziwen Tang, Ruoni Lin, Zhou Liang, Feng Li, Changjian Zhu, Xu Han, Ruilin Zheng, Jiani Shen, Jing Yu, Ning Luo, Wenxing Peng, Jiaqing Tan, Xiaoyan Li, Jinjin Fan, Qiong Wen, Xin Wang, Jianbo Li, Xunhua Zheng, Qinghua Liu, Jianping·Guo, Guo-Ping Shi, Haiping Mao, Wei Chen, Sheng Yin*, Yi Zhou**

The Supplementary Materials include:

Figure S1. The effects of diterpene compounds on cell viability.
Figure S2. **DA** inhibited the activation of fibroblasts.
Figure S3. **DA** inhibited β-catenin pathway instead of TGF-β/Smad signaling.
Figure S4. Thermal-shifted proteins identified by TPP.
Figure S5. **DA** inhibited Cdc42 activity.
Figure S6. ZCL278 exhibited systemic and hepatic toxicity compared to **DA**.
Figure S7. Knockdown of Cdc42 inhibited fibrosis induced by Ang II, IL-17, LPS, and IL-1β.
Figure S8. **DA** targets Cdc42 to regulate macrophages and T cell functions.
Table S1. Diterpenoids isolated from *W. chamaedaphne*.
Table S2. Possible protein targets of **DA**.
Table S3. Antibodies used for immunoblotting.
Table S4. Primes used for q-PCR experiments.
Table S5. si-RNAs used for transfection experiments.
Table S6. Clinical characteristics for patient samples included in this study.

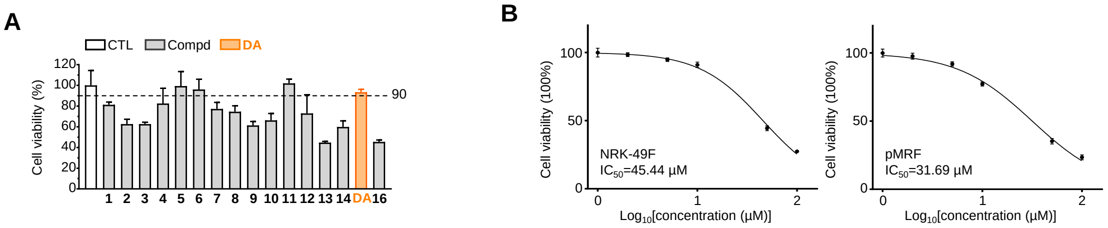

Figure S1. The effects of diterpene compounds on cell viability. A) MTS assay of the influence of diverse diterpene compounds (Compd) (10 μM) on cell proliferation of NRK-49F cells for 2 days (*n*=6 per group); B) Cell viability curves of NRK-49F cell line or pMRF treated with **DA** of indicated concentrations for 48 h, measured by MTS assay (*n*=6 per group). pMRF, primary murine renal fibroblast.

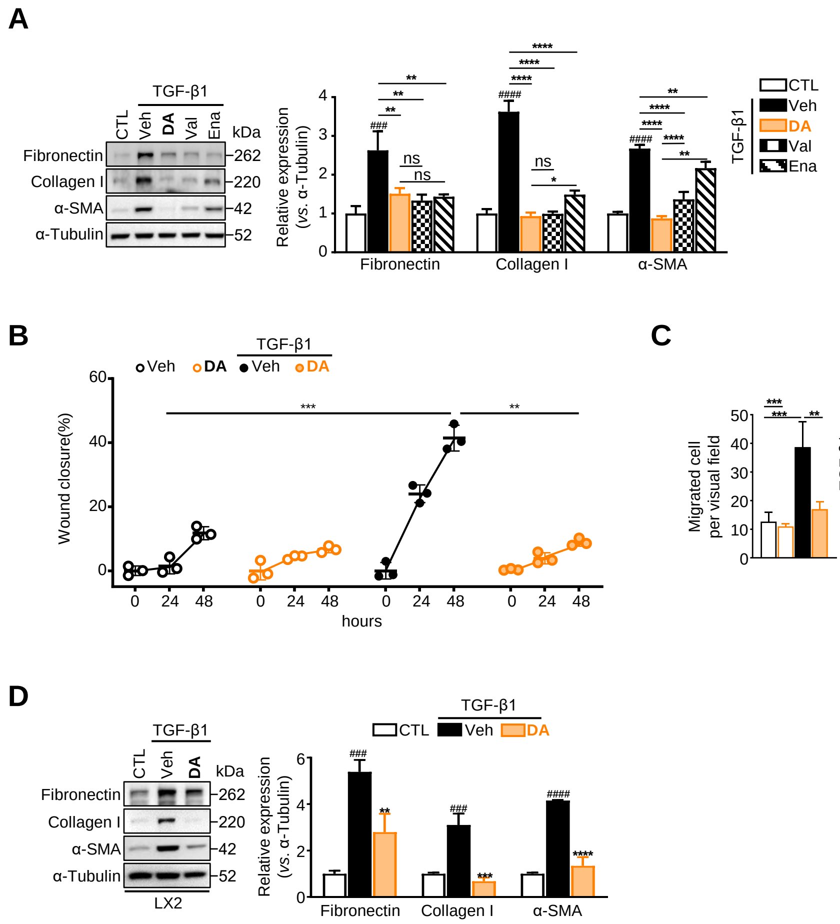

Figure S2. DA inhibited the activation of fibroblasts. A) Western blots performed on TGF-β1-activated NRK-49F following the treatment of **DA** (5µM), valsartan (Val, 5 µM), enalapril (Ena, 5 µM) (*n*=3 per group). B) Statistical analysis of wound healing assay (*n*=3 per group). C) Statistical analysis of the transwell assay (*n*=3 per group). D) Immunoblot analysis of fibronectin, collagen I, and α-SMA in TGF-β1-stimulated LX2 cells treated with vehicle or **DA** (*n*=3 per group). Control, CTL; vehicle, Veh. Data are presented as means ± SEM. Statistical analysis was performed using one-way ANOVA followed by a Bonferroni's multiple comparisons test. ### $P < 0.001$, #### $P < 0.0001$ compared with CTL group. * $P < 0.05$, ** $P < 0.01$, **** $P < 0.0001$ compared with Veh group or groups under the lines; ns, not significant..

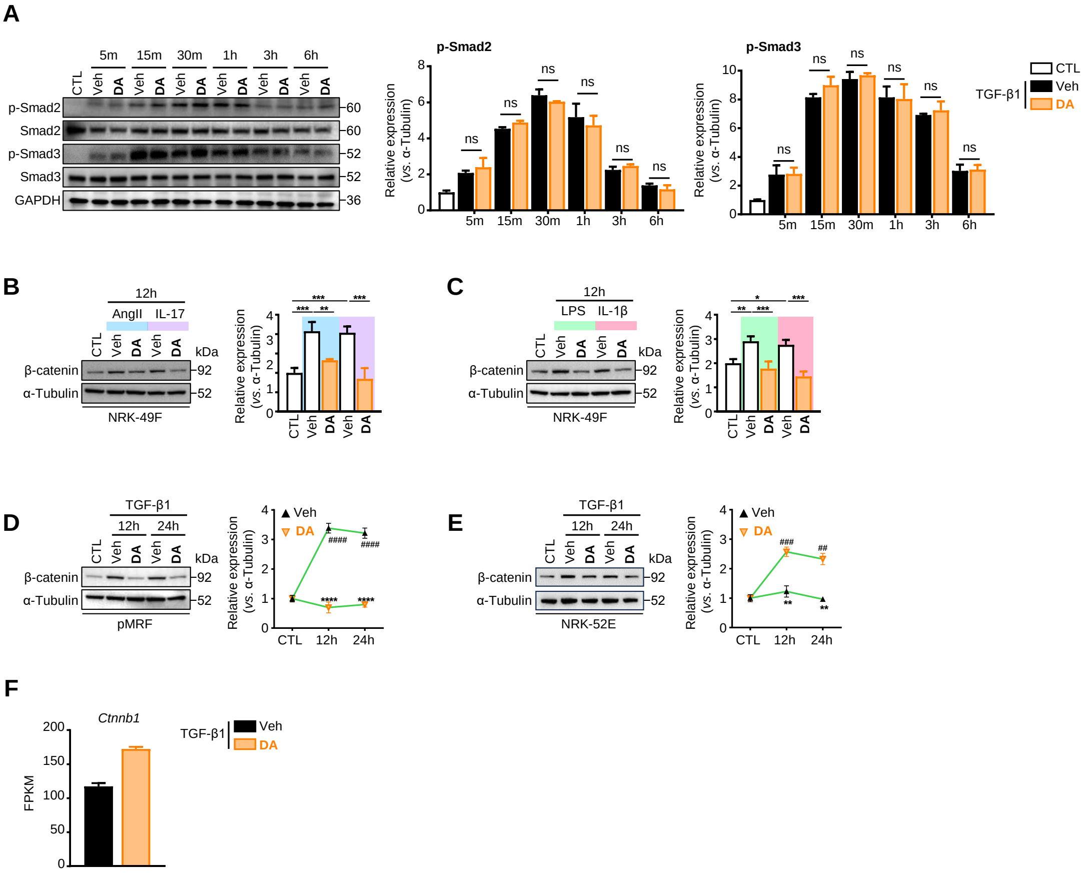

Figure S3. DA inhibited β-catenin pathway instead of TGF-β/Smad signaling. A) Protein levels of phosphorylated Smad2 and Smad3 (also p-Smad2 and p-Smad3) were detected at indicated times in TGF-β1-stimulated NRK-49F treated with vehicle or **DA** (*n*=3 per group). B, C) Immunoblots showing the effects of **DA** on β-catenin in NRK-49F cells incubated with (B) AngII (10μM) and IL-17 (50 ng/mL), (C) LPS (25 ng/mL) and IL-1β (10 ng/mL) (*n*=3 per group). D, E) **DA**'s effects on β-catenin in (D) pMRF (*n*=3 per group) and (E) NRK-52E (*n*=3 per group); F) The FPKM value of the transcript of β-catenin (Ctnnb1) in TGF-β1-activated NRK-49F cells treated with vehicle or **DA** (*n*=3 per group) (student's t-test). Fragments per kilobase per million, FPKM; Control, CTL; vehicle, Veh; ns, not significant. Data are presented as means ± SEM and were analyzed using one-way ANOVA followed by Bonferroni's multiple comparisons test unless otherwise stated. $^{##}$ $P < 0.01$, $^{####}$ $P < 0.0001$ compared with CTL group; * $P < 0.05$, ** $P < 0.01$, *** $P < 0.001$, **** $P < 0.0001$ compared with Veh group or between groups under the line.

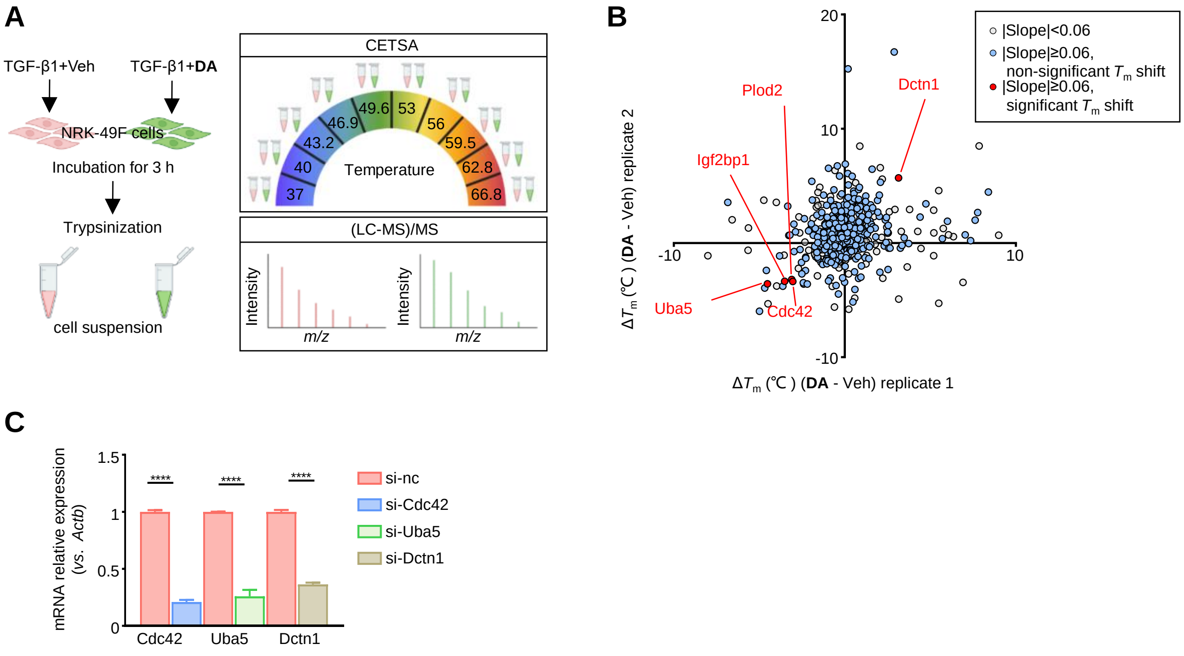

Figure S4. Thermal-shifted proteins identified by TPP. A) Flow chart of TPP. NRK-49F cells were incubated with vehicle (DMSO) or **DA** for 3 h. Subsequently, cells were trypsinized into cell suspension. Cellular thermal shift assay (CETSA) was then performed. For each condition, the cell sample was divided into 10 aliquots. Aliquots were subjected to heating at the indicated temperatures. All samples from each condition were analyzed utilizing LC-MS/MS. The obtained reporter ion intensities were used to fit a melting curve and calculate the melting temperature (T_m) of each protein separately for the two conditions. B) According to TPP results, a flat slope of the melting curve relates to lower T_m reproducibility, thus proteins with an absolute slope below 0.06 were excluded and plotted in gray. Proteins with an absolute slope above 0.06 were plotted in blue and their Tm shifts were further evaluated. Criteria for a significant Tm shift were listed as follows: 1) melting point shifts in the two **DA** vs DMSO experiments have the same sign; 2) both the melting point differences (ΔT_m) in the **DA** vs DMSO experiments are greater than the ΔT_m between the two DMSO experiments. **DA**-induced T_m shifts that passed the significance criteria are shown in red. C) Effects of si-Cdc42, si-Uba5, or si-Dctn1 on Cdc42, Uba5, and Dctn1 mRNA expression levels respectively. β-actin (*Actb)* was used as the internal reference (*n*=3 per group). Nc, negative control. Data were presented as means ± SEM and analyzed using student t-test. **** $P <$ 0.0001 compared with si-nc group.

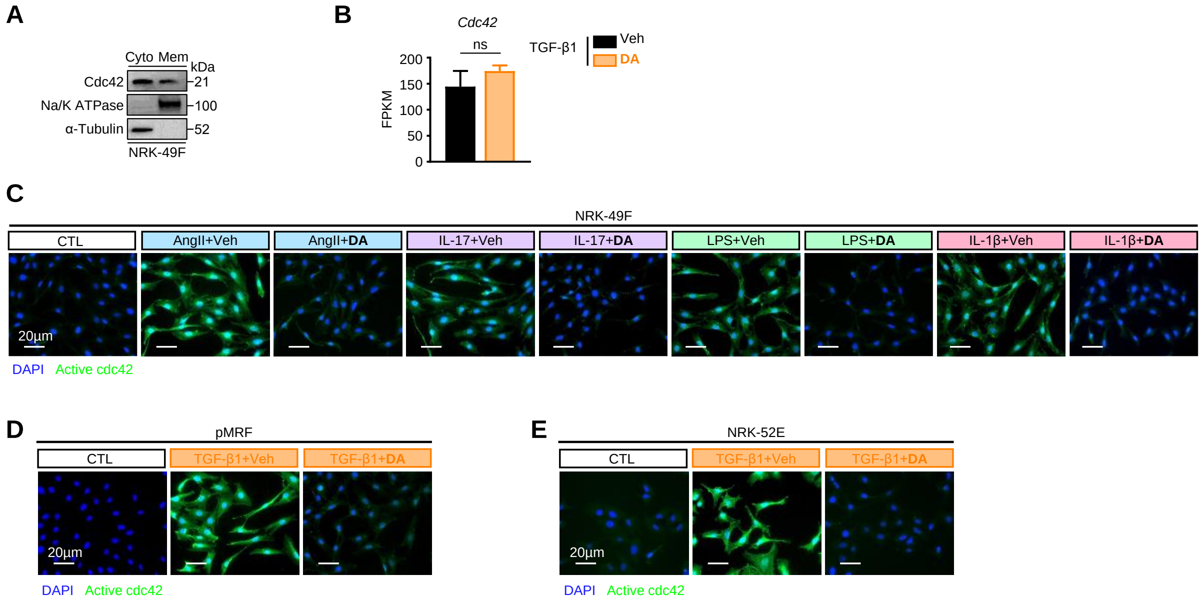

Figure S5. DA inhibited Cdc42 activity. A) Immunoblots of Cdc42 in cytoplasm and membrane of NRK-49F. B) The FPKM value of the transcript of Cdc42 (*Cdc42*) in TGF-β1-activated NRK-49F cells treated with vehicle or **DA** (*n*=3 per group). C, D, E) Representative immunofluorescence microscopy of active Cdc42. (C)Serum-starved NRK-49F cells were stimulated by AngII (10μM), IL-17 (50 ng/mL), LPS (25 ng/mL), and IL-1β (10 ng/mL) and treated with vehicle or 5 μM **DA**; (D) pMRF and (E) NRK-52E were activated by TGF-β1 and treated with vehicle or 5 μM **DA**. Cells were probed with an active Cdc42 antibody (green). Nuclei were visualized by DAPI (blue). Scale bar = 20 μm. Fragments per kilobase per million, FPKM; Vehicle, Veh. Data in B were presented as means ± SEM and analyzed using student t-test.

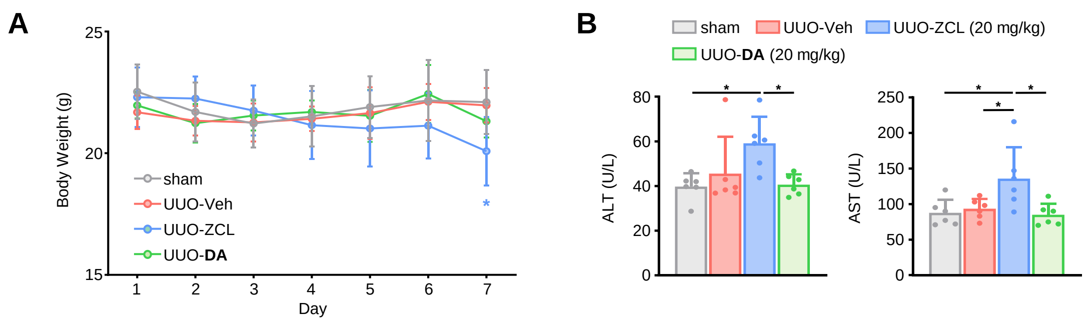

Figure S6. ZCL278 exhibited systemic and hepatic toxicity compared to **DA**. A) Mice body weights in the 7 consecutive days after sham or UUO surgery (n=6 per group). B) The levels of serum AST and ALT of **DA** or ZCL-treated mice were measured at day 7 after the surgery (n=6 per group). Data were analyzed by two-way ANOVA followed by Bonferroni's multiple comparisons test. Vehicle, Veh; low dose, LD; high dose, HD; ZCL, ZCL278. Data are presented as means ± SEM. * $P < 0.05$.

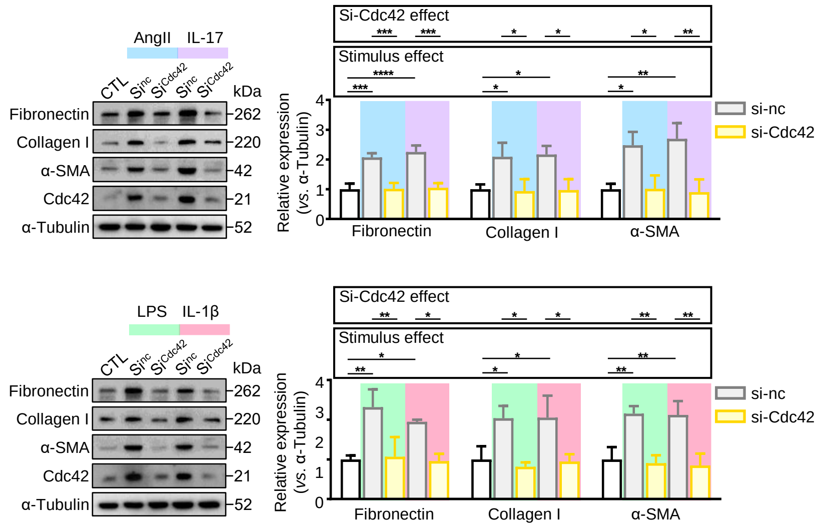

Figure S7. Knockdown of Cdc42 inhibited fibrosis induced by Ang II, IL-17, LPS, and IL-1β. NRK-49F cells were transfected with si-Cdc42 or negative control siRNA (si-nc) and incubated with AngII (10μM), IL-17 (50 ng/mL), LPS (25 ng/mL), and IL-1β (10 ng/mL) for 48 hours (*n*=3 per group). Fibrotic markers were detected by immunoblotting. Data are presented as means ± SEM and were analyzed using one-way ANOVA followed by Bonferroni's multiple comparisons test unless otherwise stated. CTL, control; nc, negative control. * $P < 0.05$, ** $P < 0.01$, *** $P < 0.001$, **** $P < 0.0001$ compared with si-nc group or between groups under the line.

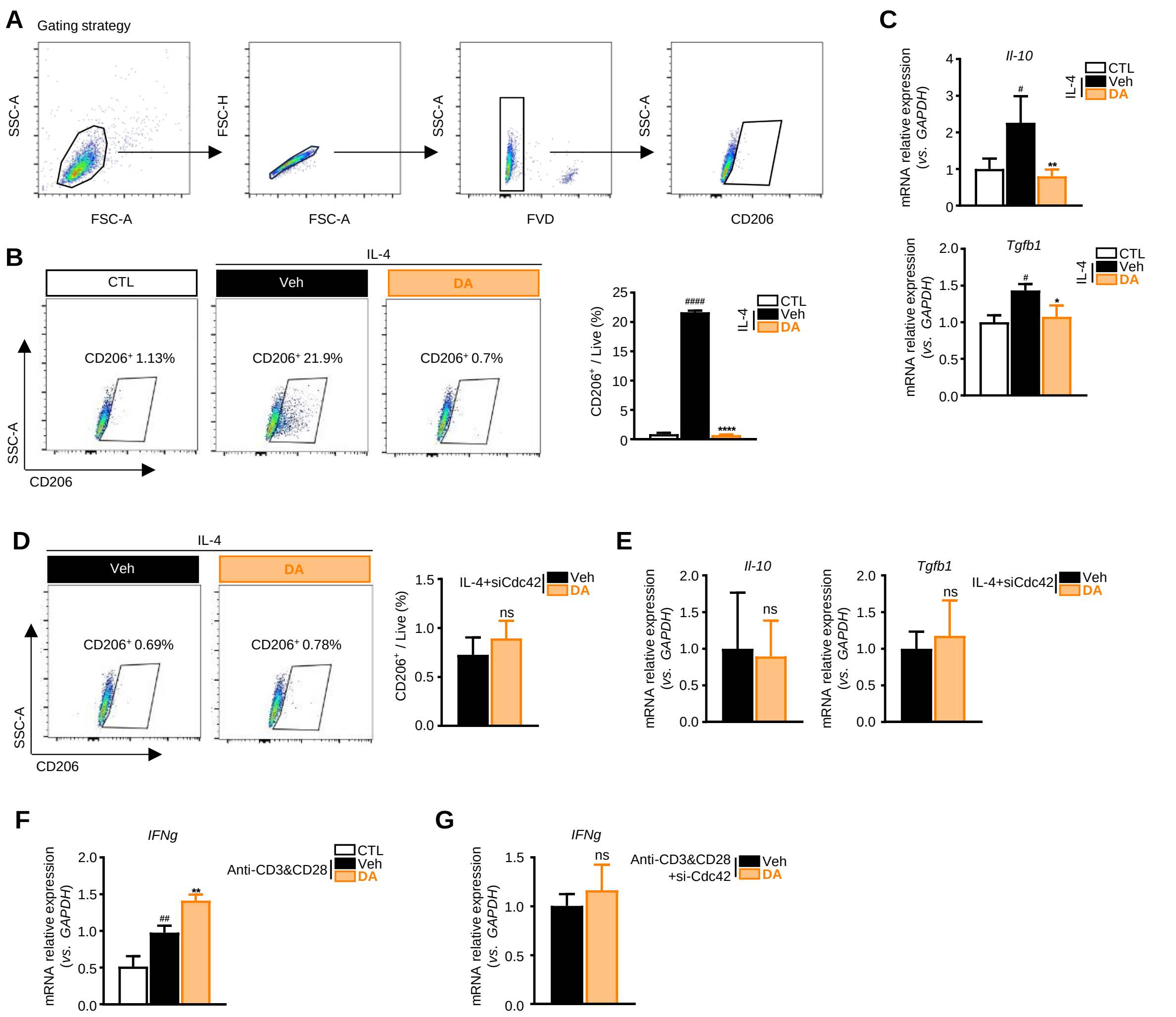

Figure S8. DA targets Cdc42 to regulate macrophages and T cell functions. A) Gating strategy used to identify M2 macrophages. B) Representative flow cytometry plots and statistical data showing the percentage of M2 macrophages ($CD206^+$) within live cells (FVD^-) cells in RAW264.7 (CTL) and RAW264.7 treated with IL-4+vehicle or IL-4+**DA** (*n*=3 per group). C) The real-time PCR analysis of IL-10 (*Il-10*) and TGFβ1 (*Tgfb1*) in RAW264.7 (CTL) and RAW264.7 treated with IL-4+vehicle or IL-4+**DA** (*n*=3 per group). D) Representative flow cytometry plots and statistical data showing the percentage of M2 macrophages ($CD206^+$) within live cells (FVD^-) cells (WBC) in Cdc42-knocked down RAW264.7treated with IL-4+vehicle or IL-4+**DA** (*n*=3 per group). E) The real-time PCR analysis of IL-10 (*Il-10*) and TGFβ1 (*Tgfb1*) in Cdc42-knocked down RAW264.7 treated with IL-4+vehicle or IL-4+**DA** (*n*=3 per group). F) The real-time PCR analysis of IFN-γ (*IFNg*) in Jurkat (CTL) and Jurkat treated with anti-CD3&CD28+vehicle or anti-CD3&CD28+**DA** (*n*=3 per group). G) The real-time PCR analysis of IFN-γ (*IFNg*) in Cdc42-knocked down Jurkat

treated with anti-CD3&CD28+vehicle or anti-CD3&CD28+**DA** (*n*=3 per group). Data are presented as means ± SEM. Student's t test (D, E, G) and one-way ANOVA test followed by Bonferroni's multiple comparisons test (B, C, F) were performed. CTL, control; nc, negative control; Veh, vehicle. $^{\#}$ $P < 0.05$, $^{\#\#}$ $P < 0.01$, $^{\#\#\#\#}$ $P < 0.0001$ compared with CTL group. * $P < 0.05$, ** $P < 0.01$, **** $P < 0.0001$ compared with vehicle group; ns, not siginificant

Table S1. Diterpenoids isolated from *W. chamaedaphne*.

Number	Name	Structure
**1**	Simplexin	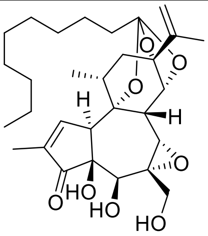
**2**	6α,7α-Epoxy-5β-hydroxy-12-deoxyphorbol-13-decanoate	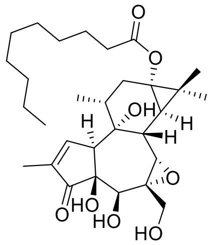
**3**	Genkwanine P	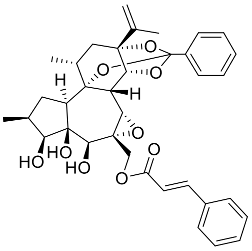
**4**	Wikstroelide W	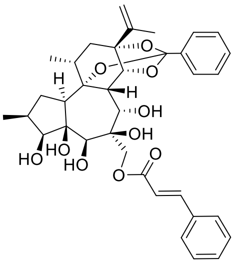
**5**	daphnetoxin	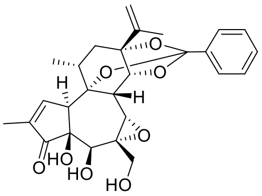
**6**	Pimelotide A	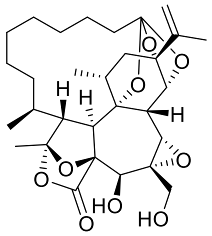

**7**	Daphnepedunin B	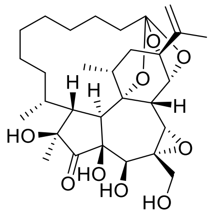
**8**	Pimelea factor S6	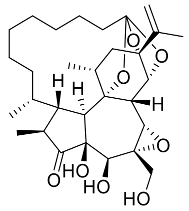
**9**	Wikstroelide E	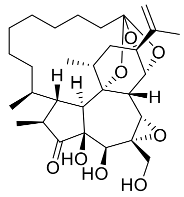
**10**	Gnidimacrin	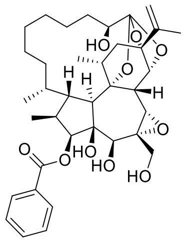
**11**	Trigochinin A	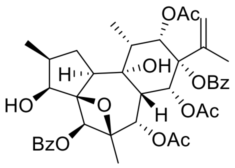
**12**	Trigochinin D	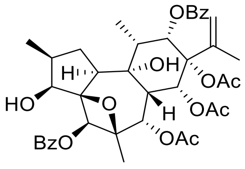
**13**	Trigolin C	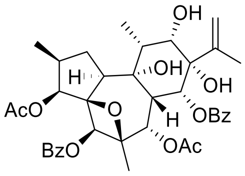
**14**	Trigolin F	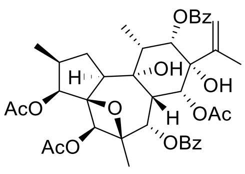

**15**	Daphnepedunin A	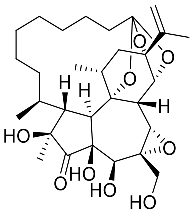
**16**	Pimelotide C	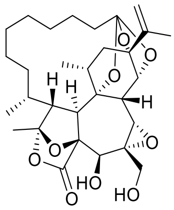

Table S2. Possible protein targets of **DA**.

UniProt ID	Protein Description	Name
Q811A3	Procollagen-lysine,2-oxoglutarate 5-dioxygenase 2	Plod2
Q8CFN2	Cell division control protein 42 homolog	Cdc42
Q8CGX0	Insulin-like growth factor 2 mRNA-binding protein 1	Igf2bp1
P28023	Dynactin subunit 1	Dctn1
Q5M7A4	Ubiquitin-like modifier-activating enzyme 5	Uba5

Table S3. Antibodies used for immunoblotting.

Name	Source	Dilution ratio	Catalog number
rabbit anti-fibronectin	Boster	1:1000	BA1772
rabbit anti-collagen I	Boster	1:1000	BA0325
mouse anti-α-SMA	Sigma	1:1000	A5228
mouse anti-E-cadherin	Abcam	1:1000	ab231303
rabbit anti-vimentin	Boster	1:1000	PB9359
rabbit anti-β-catenin	CST	1:1000	8480
rabbit anti-phospho-β-catenin (Ser33/34/Thr41)	CST	1:1000	9561
rabbit anti-phospho-β-catenin (Thr41/Ser45)	CST	1:1000	9565
rabbit anti-phospho-β-catenin (Ser552)	CST	1:1000	5651
rabbit anti-phospho-β-catenin (Ser675)	CST	1:1000	4176
rabbit anti-phospho-GSK-3β (Ser9)	CST	1:1000	9336
rabbit anti-phospho-PKCζ (Thr410/403)	CST	1:1000	9378
rabbit anti-Smad2	CST	1:1000	5339
rabbit anti-phospho-Smad2	CST	1:1000	3108
rabbit anti-Smad3	CST	1:1000	9523
rabbit anti-phospho-Smad3	CST	1:1000	9520
mouse anti-Cdc42	Santa Cruz	1:100	sc-8401
rabbit anti-Lamin b1	CST	1:1000	12586
rabbit anti-Na/K-ATPase	CST	1:1000	3010
Mouse anti-His	CST	1:1000	2366
HRP conjugated rabbit anti-α-Tubulin	CST	1:2000	12351
HRP conjugated goat anti-rabbit IgG	CST	1:10000	7074
HRP conjugated goat anti-rabbit IgG	CST	1:10000	91196

Table S4. Primes used for q-PCR experiments.

Gene	Genus	Sequnce
Cdc42	Rat	F: 5’-GCGGAGAAGCTGAGGTCAA-3’
		R: 5’-TGGTACATACTCCGACGGGA-3’
Uba5	Rat	F: 5’-GTGAGCCCAGCAGAGCAG-3’
		R: 5’-ACAGCATAGGTACGGATTTTCTCA -3’
Dctn1	Rat	F: 5’-AAGGCCACGGCATCTTTGTA-3’
		R: 5’-TCGAGTTGTGGTCTTTCGGG-3’
Actb	Rat	F: 5’-AGGTCATCACTATCGGCA-3’
		R: 5’-CCACCAATCCACACAGAG-3’
Acta2	Mouse	F: 5’-GGCTGTATTCCCCTCCATCG-3’
		R: 5’-CCAGTTGGTAACAATGCCATGT-3’
Col1a1	Mouse	F: 5’-GATGACGTGCAATGCAATGAA-3’
		R: 5’-CCCTCGACTCCTACATCTTCTGA-3’
Col3a1	Mouse	F: 5’-GAAAGAGGATCTGAGGGCTCG-3’
		R: 5’-GGGTGAAAAGCCACCAGACT-3’
Fn1	Mouse	F: 5’-TACCAAGGTCAATCCACACCCC-3’
		R: 5’-CAGATGGCAAAAGAAAGCAG-3’
Actb	Mouse	F: 5’-GGCTGTATTCCCCTCCATCG-3’
		R: 5’-CCAGTTGGTAACAATGCCATGT-3’
Il10	Mouse	F: 5’-CAGGGATCTTAGCTAACGGAAA-3’
		F: 5’-GCTCAGTGAATAAATAGAATGGGAAC-3’
Tgfb1	Mouse	F: 5’-CAACCCAGGTCCTTCCTAAA-3’
		F: 5’-GGAGAGCCCTGGATACCAAC-3’
Cdc42	Mouse	F: 5’-TGTTGGTGATGGTGCTGTTG-3’
		R: 5’-TCTCAGGCACCCACTTTTCT-3’
IFNg	Human	F: 5’-ATCGTTTTGGGTTCTCTTGGC-3’
		F: 5’-TTTTTCTGTCACTCTCCTCTTTCC-3’
CDC42	Human	F: 5’-AACGTGAAAGAAAAGTGGGTGC-3’
		F: 5’-CTTCCTTTTGGGTTGAGTTTCCG-3’
GAPDH	Human	F: 5’-GTGGACCTGACCTGCCGTCT-3’
		F: 5’-GGAGGAGTGGGTGTCGCTGT-3’

Table S5. si-RNAs used for transfection experiments.

Target gene	Sequence
Cdc42 (Rat)	5′-GCAAGAGGAUUAUGACAGATT UCUGUCAUAAUCCUCUUGCTT-3′
Cdc42 (Mouse)	5′-GUCCAAAGACUCCUUUCUUTT AAGAAAGGAGUCUUUGGACTT-3′
Cdc42 (Human)	5′-CUGCAGGGCAAGAGGAUUATT UAAUCCUCUUGCCCUGCAGTT-3′
Uba5 (Rat)	5′-GGUCAGACAUGGAUGGAAUTT AUUCCAUCCAUGUCUGACCTT-3′
Dctn1 (Rat)	5′-GCUCAGAAGACAAAGCAAATT UUUGCUUUGUCUUCUGAGCTT-3′
Control	5′-UUCUCC GAACGUGUCACGUTTACGUGACACGUUCGGAGAATT-3′

Table S6. Clinical characteristics for patient samples included in this study.

Age [year]	Sex	Diagnosis	Scr[a)] [μmol/L]	Urea [mmol/L]	eGFR[b)] [mL/min/1.73m^2]
55	Male	Focal segmental glomerulosclerosis	172	10.5	37.72
39	Male	Hypertensive nephropathy	131	5.4	58.67
34	Male	Lupus nephritis	346	27.3	18.78
35	Male	Focal segmental glomerulosclerosis	67	4.1	118.33
71	Male	Nephrotic syndrome	230	26.4	23.73
28	Female	IgA nephropathy	99	5.9	66.8
25	Female	Lupus nephritis	79	7.9	89.63
41	Female	Lupus nephritis	131	9.1	43.46
59	Female	Nephrotic syndrome	622	18.1	5.82
33	Male	Focal segmental glomerulosclerosis	92	3.8	93.81
56	Female	Nephrotic syndrome	277	14.8	15.82
37	Male	Nephrotic syndrome	124	8.1	63.58

[a)]Scr, serum creatine; [b)]eGFR, estimated glomerular filtration rate
